# A systematic review of radiomics in pancreatitis: applying the evidence level rating tool for promoting clinical transferability

**DOI:** 10.1186/s13244-022-01279-4

**Published:** 2022-08-20

**Authors:** Jingyu Zhong, Yangfan Hu, Yue Xing, Xiang Ge, Defang Ding, Huan Zhang, Weiwu Yao

**Affiliations:** 1 Department of Imaging, Tongren Hospital, Shanghai Jiao Tong University School of Medicine, No. 1111 Xianxia Road, Shanghai, 200336 China; 2 Department of Radiology, Ruijin Hospital, Shanghai Jiao Tong University School of Medicine, No. 197 Ruijin 2nd Road, Shanghai, 200025 China

**Keywords:** Pancreatitis, Machine learning, Differential diagnosis, Quality improvement, Systematic review

## Abstract

**Background:**

Multiple tools have been applied to radiomics evaluation, while evidence rating tools for this field are still lacking. This study aims to assess the quality of pancreatitis radiomics research and test the feasibility of the evidence level rating tool.

**Results:**

Thirty studies were included after a systematic search of pancreatitis radiomics studies until February 28, 2022, via five databases. Twenty-four studies employed radiomics for diagnostic purposes. The mean ± standard deviation of the adherence rate was 38.3 ± 13.3%, 61.3 ± 11.9%, and 37.1 ± 27.2% for the Radiomics Quality Score (RQS), the Transparent Reporting of a multivariable prediction model for Individual Prognosis Or Diagnosis (TRIPOD) checklist, and the Image Biomarker Standardization Initiative (IBSI) guideline for preprocessing steps, respectively. The median (range) of RQS was 7.0 (− 3.0 to 18.0). The risk of bias and application concerns were mainly related to the index test according to the modified Quality Assessment of Diagnostic Accuracy Studies (QUADAS-2) tool. The meta-analysis on differential diagnosis of autoimmune pancreatitis versus pancreatic cancer by CT and mass-forming pancreatitis versus pancreatic cancer by MRI showed diagnostic odds ratios (95% confidence intervals) of, respectively, 189.63 (79.65–451.48) and 135.70 (36.17–509.13), both rated as weak evidence mainly due to the insufficient sample size.

**Conclusions:**

More research on prognosis of acute pancreatitis is encouraged. The current pancreatitis radiomics studies have insufficient quality and share common scientific disadvantages. The evidence level rating is feasible and necessary for bringing the field of radiomics from preclinical research area to clinical stage.

**Supplementary Information:**

The online version contains supplementary material available at 10.1186/s13244-022-01279-4.

## Key points

- More high-quality research on prognosis of acute pancreatitis is encouraged, since it has great influence on clinical decision-making but cannot be easily predicted by radiologists’ assessment.
- The overall RQS rating could detect common methodological issues across radiomics research, but the biological correlation and comparison to “gold standard” item needs further modification for non-oncological radiomics studies.
- The RQS rating, TRIPOD checklist, and IBSI for preprocessing steps can serve as tools for radiomics quality evaluation in non-oncological field, while the development of a single comprehensive tool is more favorable for future evaluation.
- An evidence level rating tool has been confirmed to be feasible for the determination of the existing gap between preclinical and clinical use of radiomics research and is necessary for the overall assessment of specific clinical problems.

## Background

Acute pancreatitis is a frequent pancreatic disease that is characterized by a local and systemic inflammatory response with the varying clinical course from self-limiting mild acute pancreatitis to moderate or severe acute pancreatitis which has a substantial mortality rate [1]. A plethora of studies attempted to predict the severity of acute pancreatitis to guide clinical treatment, such as the Acute Physiology and Chronic Health Evaluation (APACHE) II [2], the bedside index for severity in acute pancreatitis (BISAP) [3], and the CT severity index (CTSI) [4]. However, complexity in evaluation may hinder their clinical application, and they are not useful for predicting recurrence or local complications [2–4]. Approximately 20% of acute pancreatitis patients endure recurrent attacks and progress to chronic pancreatitis, a fibroinflammatory syndrome of the exocrine pancreas [5]. Chronic pancreatitis may present mass-like or cyst-like appearance, mimicking mass-forming pancreatitis, autoimmune pancreatitis, pancreatic cancer, and other pancreatic tumors [6]. The differential diagnosis and determination of malignancy of these lesions are hard, but it is necessary to achieve an accurate diagnosis to avoid unnecessary surgery in inflammatory conditions.

Radiomics represents the process of extracting quantitative features to transform images into high-dimensional data for capturing deeper information to support decision-making [7–11]. Current studies have shown its potential for pancreatic precision medicine, especially in diagnosis and management of pancreatic tumors [12–14]. Although the main use of radiomics lies in oncology, the radiomics approach is suitable for non-oncological research based on its nature [15–17]. However, only 5.6% of pancreatic radiomics studies investigated the role of radiomics in acute pancreatitis [18]. Most radiomics studies on chronic, mass-forming, or autoimmune pancreatitis were aimed to differentiate these inflammatory conditions from malignancy lesions [19–22]. Implanting radiomics in acute pancreatitis could provide predictive information to identify patients with worse prognosis and therefore promote personalized medical treatment. It is also important to identify patients with a high risk of chronic pancreatitis to allow for closer follow-up and early intervention. Further, the current radiomics reviews applied multiple tools for quality assessment, while the study quality and clinical value of radiomics in pancreatitis are unknown. A high level of evidence is an essential prerequisite for translating radiomics into clinical use. To the best of our knowledge, the level of evidence supporting radiomics models for clinical practice has not been fully investigated.

Hence, our review is aimed to systematically evaluate the methodology quality, reporting transparency, and risk of bias of current radiomics studies on pancreatitis, and determine their level of evidence according to the results of meta-analyses.

## Methods

### Protocol and registration

The protocol of the current systematic review has been drafted and registered (Additional file 1: Note S1). This systematic review followed the Preferred Reporting Items for Systematic Reviews and Meta-analysis (PRISMA) statement [23], and the relevant checklists are available as Additional file 2.

### Literature search and study selection

A systematic search of articles on radiomics in pancreatitis was performed via PubMed, Embase, Web of Science, China National Knowledge Infrastructure, and Wanfang Data until February 28, 2022, with a search string combining “radiomics” and “pancreatitis.” There was no limitation of publish period, but only articles written in English, Chinese, Japanese, German or French were eligible. The reference lists of included articles and relevant reviews were screened to identify additional eligible articles. We included primary radiomics articles whose purposes were diagnostic, prognostic, or predictive. Two reviewers each with 4 years of experience in radiomics and systematic review searched and selected articles independently. In case of disagreements, a third reviewer with 30 years of experience in abdominal radiology and experience in radiomics research would be consulted. The detailed search strategy and eligibility criteria are available in Additional file 1: Note S2.

### Data extraction and quality assessment

We modified a data extraction sheet for the current review, which includes literature information, study characteristics, radiomics considerations, and model metrics (Additional file 1: Table S1) [24]. One reviewer extracted the data independently and then the other reviewer cross-checked the results. The disagreements were resolved by a third reviewer.

The Radiomics Quality Score (RQS) [10], the Transparent Reporting of a multivariable prediction model for Individual Prognosis Or Diagnosis (TRIPOD) checklist [25], the Image Biomarker Standardization Initiative (IBSI) guideline [11], and the modified Quality Assessment of Diagnostic Accuracy Studies (QUADAS-2) tool [26] were employed to assess the study quality (Additional file 1: Tables S2 to S5). These tools were modified to current review topic. Briefly, the RQS with 16 items was used to assess the methodological quality of radiomics according to six key domains [27]. The TRIPOD was partially modified into a 35-item checklist for application in radiomics, excluding the Additional file 1 and funding items [28]. Due to the overlapping with the RQS and the TRIPOD, only seven items relevant to preprocessing steps were selected from the IBSI guideline [29]. The QUADAS-2 tool was tailored to the current research question through signaling questions for risk of bias and application concerns [24]. Two reviewers rated the articles independently, and the disagreements were resolved by discussion with a third reviewer. The consensus reached during data extraction and quality assessment is described in Additional file 1: Note S3.

### Data synthesis and analysis

The characteristics of included studies were descriptively summarized. The RQS score and the percentage of the ideal score were described as the mean score and the percentage of mean score to ideal score for each item, respectively. The adherence rates of the RQS rating, the TRIPOD checklist and the IBSI guideline were calculated as the ratio of the number of articles with basic adherence to the number of all available articles. In case a score of at least one point for each item was obtained without minus points, it was considered to have basic adherence, as those which have been reported [27–29]. During the calculation of TRIPOD, the “if done” or “if relevant” items (5c, 11, and 14b) and validation items (10c, 10e, 12, 13, 17, and 19a) were excluded from both the denominator and numerator [28, 29]. The result of QUADAS-2 assessment was summarized as proportions of high risk, low risk and unclear.

Subgroup analysis was performed to determine whether a factor influenced on the ideal percentage of RQS, the TRIPOD adherence rate, and the IBSI adherence rate, including the journal type, first authorship, biomarker, and imaging modality. According to the data distribution, Student’s *t* test or Mann–Whitney’s *U* test was used for intergroup differences, and one-way analysis of variance or Kruskal–Wallis *H* test was applied for multiple comparisons. The Spearman correlation test was used for the correlation analysis between the study quality (the ideal percentage of RQS, the TRIPOD adherence rate, and the IBSI adherence rate) and characteristics (the sample size and the impact factor). The SPSS software version 26.0 was used for statistical analysis. A two-tailed *p* value < 0.05 was recognized as statistical significance, unless otherwise specified.

In the current review, the value of radiomics in differential diagnosis of autoimmune pancreatitis versus pancreatic cancer by CT and mass-forming pancreatitis versus pancreatic cancer by MRI were repeatedly addressed. Therefore, these two clinical questions were included in the meta-analysis. We performed meta-analysis according to imaging modalities, to present the clinically practicable estimation. One reviewer directly extracted or reconstructed the two-by-two tables based on available data, and then the other reviewer cross-checked the results. The diagnostic odds ratio (DOR) with its 95% confidence interval (CI) and the corresponding *p* value were calculated using random effect model. The sensitivity, specificity, positive and negative likelihood ratio and their 95% CIs were also quantitatively synthesized. The hierarchical summary receiver operating characteristic (HSROC) curve was drawn for visual evaluation of diagnostic performance and heterogeneity. The Cochran’s Q test and the Higgins I^2^ test were conducted for heterogeneity assessment. The Deeks funnel plot was constructed for publication bias. The Deeks funnel asymmetry test, Egger’s test, and Begg’s test were performed. A two-tailed *p* value > 0.10 indicated a low publication bias. The trim and fill method was employed to evaluate the robustness of meta-analyses. The Stata software version 15.1 with metan, midas, and metandi packages was employed for meta-analysis.

The model type and phase of image mining studies of the studies were classified according to the TRIPOD statement (Additional file 1: Table S6) [25] and a previous review (Additional file 1: Table S7) [30]. The levels of evidence supporting clinical values were rated based on the results of meta-analyses (Additional file 1: Table S8) [31, 32]. The detailed analysis methods are described in Additional file 1: Note S4.

## Results

### Literature search

**Fig. 1:**
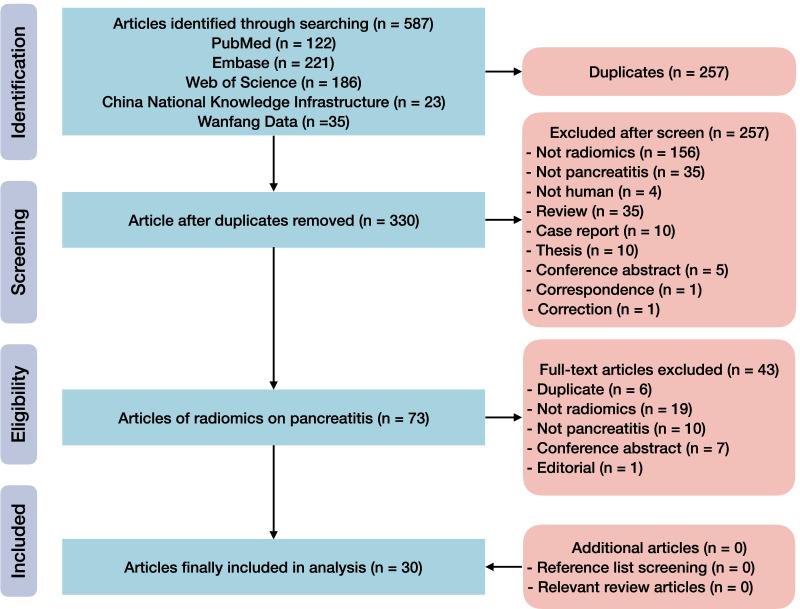
Flow diagram of study inclusion

The search identified 587 records in total, 257 of which were excluded due to duplication. After screening the remaining 330 records, 73 full texts were retrieved and reviewed. Finally, 30 studies were included (Fig. 1) [33–62]. No additional eligible study was found through hand search of their reference lists or relevant reviews.

### Study characteristics

**Fig. 2:**
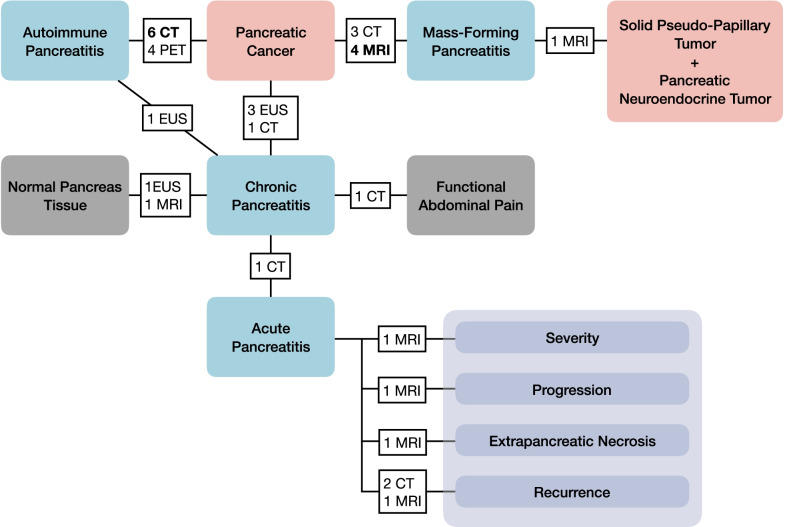
Study topics and number of studies. Three studies investigated two topics, respectively, and had been treated as two different studies in the term of topic. Therefore, there were thirty studies according to article, but thirty-three models according to topic. The bolded number with modality indicates the studies included in the meta-analysis

The characteristics of the 30 included studies are summarized in Table 1. Figure 2 shows the topics of the 33 models included in the 30 studies. 69.7% (23/33) models focused on the role of radiomics in differential diagnosis of pancreatitis from pancreatic tumors, while 12.1% (4/33) models employed radiomics to distinguish chronic pancreatitis from normal pancreas tissue, functional abdominal pain, and acute pancreatitis. The remaining 18.1% (6/33) models investigated the predictive potential of radiomics in prognosis of acute pancreatitis. The literature information, model characteristics, and radiomics information of each study are present in Additional file 1: Tables S9 to S11.

**Table 1:** Study characteristics

Study characteristics	Data
Sample size, mean ± standard deviation, median (range)	137.5 ± 85.0, 111 (41–389)
Journal type, *n* (%)	*N* = 30
Imaging	16 (53)
Non-imaging	14 (47)
First authorship, *n* (%)	*N* = 30
Radiologist	24 (80)
Non-radiologist	6 (20)
Biomarker, *n* (%)	*N* = 30
Diagnostic	24 (80)
Prognostic	6 (20)
Imaging modality, *n* (%)	*N* = 30
CT	13 (43)
EUS	4 (13)
MRI	9 (30)
PET	4 (13)
Model type, *n* (%)	*N* = 30
Type 1a: Developed model validated with exactly the same data	7 (23)
Type 1b: Developed model validated with resampling data	10 (33)
Type 2a: Developed model validated with randomly splitting data	12 (40)
Type 2b: Developed model validated with non-randomly splitting data	1 (3)
Type 3: Developed model validated with separate data	0 (0)
Type 4: Validation only	0 (0)
Phase classification, *n* (%)	*N* = 30
Phase 0: < 100 patients; retrospective; internal validation	16 (53)
Phase I: < 100 patients; retrospective; external validation	2 (7)
Phase II: > 100 patients; retrospective; external validation	12 (40)
Phase III: > 100 patients; prospective; external validation	0 (0)
Phase IV: real-world	0 (0)

### Study quality

**Fig. 3:**
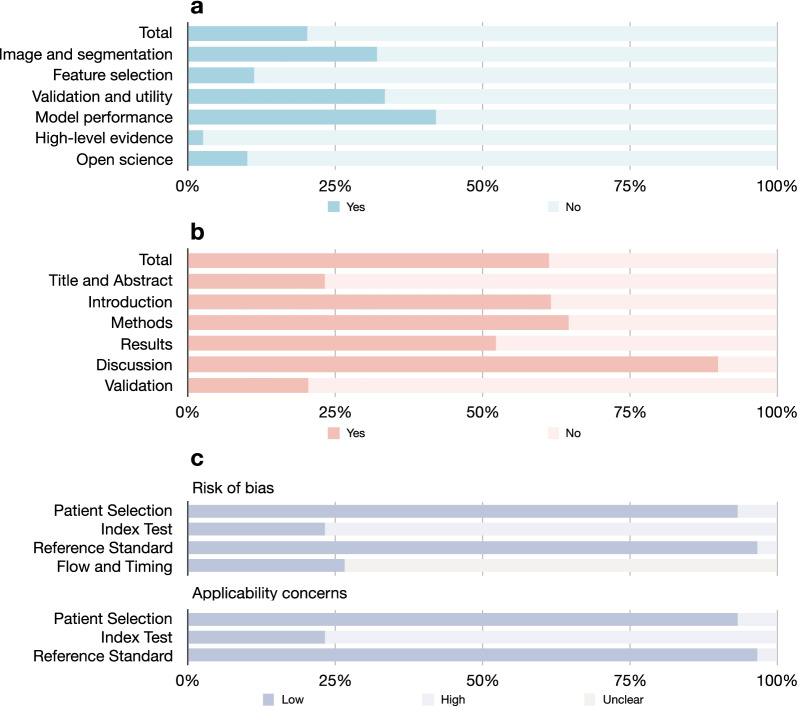
Quality assessment of included studies. **a** Ideal percentage of RQS; **b** TRIPOD adherence rate; **c** QUADAS-2 assessment result

The overall mean ± standard deviation (median, range) of the RQS rating was 7.0 ± 5.0 (7.0, − 3.0 to 18.0), with an overall adherence rate of 38.3% (184/480), and an ideal percentage of RQS of 20.3% (7.3/36) (Table 2; Fig. 3). Although more than nine-tenths of the studies performed feature reduction steps and reported discrimination statistics, none of the studies conducted test–retest analysis, phantom study, cutoff analysis, or cost-effectiveness analysis. All six key domains of RQS were suboptimal, among which the model performance index domain showed the highest ideal percentage of 42.7% (2.1/5).

**Table 2:** RQS rating of included studies

16 items according to 6 key domains	Range	Median (range)	Percentage of ideal score, *n* (%)	Adherence rate, *n* (%)
Total 16 items	− 8 to 36	7 (− 3 to 18)	7.3 (20.2)	184 (38)
Domain 1: protocol quality and stability in image and segmentation	0–5	2 (0–2)	1.6 (31.3)	47 (15)
Protocol quality	0–2	1 (0–1)	0.9 (46.7)	28 (93)
Multiple segmentations	0–1	1 (0–1)	0.6 (63.3)	19 (63)
Test–retest	0–1	0 (0–0)	0 (0)	0 (0)
Phantom study	0–1	0 (0–0)	0 (0)	0 (0)
Domain 2: feature selection and validation	− 8 to 8	− 2 (− 8 to 6)	0.9 (10.8)	42 (70)
Feature reduction or adjustment of multiple testing	− 3 to 3	3 (− 3 to 3)	2.8 (93.3)	29 (97)
Validation	− 5 to 5	− 5 (− 5 to 3)	− 1.9 (0)	13 (43)
Domain 3: biologic/clinical validation and utility	0–6	1.5 (0–6)	2.0 (33.9)	47 (39)
Non-radiomics features	0–1	0.5 (0–1)	0.5 (50.0)	15 (60)
Biologic correlations	0–1	1 (0–1)	0.6 (60.0)	18 (60)
Comparison with “gold standard”	0–2	0 (0–2)	0.8 (40.0)	12 (40)
Potential clinical utility	0–2	0 (0–2)	0.1 (6.7)	2 (7)
Domain 4: model performance index	0–5	2 (1–4)	2.1 (42.7)	34 (38)
Cutoff analysis	0–1	0 (0–0)	0 (0)	0 (0)
Discrimination statistics	0–2	2 (1–2)	1.9 (95.0)	30 (100)
Calibration statistics	0–2	0 (0–2)	0.2 (11.7)	4 (13)
Domain 5: high level of evidence	0–8	0 (0–7)	0.2 (2.9)	1 (2)
Prospective study	0–7	0 (0–7)	0.2 (3.3)	1 (3)
Cost-effectiveness analysis	0–1	0 (0–0)	0 (0)	0 (0)
Domain 6: open science and data	0–4	0 (0–1)	0.4 (10.8)	13 (43)

*RQS* Radiomics Quality Score

The overall adherence rate of the TRIPOD checklist was 61.3% (478/780), excluding “if relevant,” “if done,” and “validation” items (5c, 11, 14b, 10c, 10e, 12, 13, 17, and 19a) (Table 3; Fig. 3). None of the studies reported the blinded method during the outcome assessment (item 6b), sample size calculation (item 8), and handling of missing data (item 9). The discussion section reached the highest adherence rate of 90.0% (81/90), while the adherence rate of the validation section was only 17.3% (9/52).

**Table 3:** TRIPOD adherence of included studies

35 Selected Items in 20 Criteria According to 6 Sections (*N* = 30)	Study, *n* (%)
*Overall (excluding 5c, 11, 14b, 10c, 10e, 12, 13, 17, and 19a)*	478 (61)
Section 1: Title and Abstract	14 (23)
1. Title—identify developing/validating a model, target population, and the outcome	2 (7)
2. Abstract—provide a summary of objectives, study design, setting, participants, sample size, predictors, outcome, statistical analysis, results, and conclusions	12 (40)
Section 2: Introduction	37 (62)
3a. Background—Explain the medical context and rationale for developing/validating the model	30 (100)
3b. Objective—Specify the objectives, including whether the study describes the development/validation of the model or both	7 (23)
Section 3: Methods	252 (65)
4a. Source of data—describe the study design or source of data (randomized trial, cohort, or registry data)	30 (100)
4b. Source of data—specify the key dates	26 (87)
5a. Participants—specify key elements of the study setting including number and location of centers	30 (100)
5b. Participants—describe eligibility criteria for participants (inclusion and exclusion criteria)	21 (70)
5c. Participants—give details of treatment received, if relevant (*N* = 6)	0 (0)
6a. Outcome—clearly define the outcome, including how and when assessed	30 (100)
6b. Outcome—report any actions to blind assessment of the outcome	0 (0)
7a. Predictors—clearly define all predictors, including how and when assessed	23 (77)
7b. Predictors—report any actions to blind assessment of predictors for the outcome and other predictors	15 (50)
8. Sample size—explain how the study size was arrived at	0 (0)
9. Missing data—describe how missing data were handled with details of any imputation method	0 (0)
10a. Statistical analysis methods—describe how predictors were handled	24 (80)
10b. Statistical analysis methods—specify type of model, all model-building procedures (any predictor selection), and method for internal validation	23 (77)
10d. Statistical analysis methods—specify all measures used to assess model performance and if relevant, to compare multiple models (discrimination and calibration)	30 (100)
11. Risk groups—provide details on how risk groups were created, if done (*N* = 0)	0 (0)
Section 4: Results	94 (52)
13a. Participants—describe the flow of participants, including the number of participants with and without the outcome. A diagram may be helpful	16 (53)
13b. Participants—describe the characteristics of the participants, including the number of participants with missing data for predictors and outcome	24 (80)
14a. Model development—specify the number of participants and outcome events in each analysis	25 (83)
14b. Model development—report the unadjusted association between each candidate predictor and outcome, if done (*N* = 5)	1 (20)
15a. Model specification—present the full prediction model to allow predictions for individuals (regression coefficients, intercept)	5 (17)
15b. Model specification—explain how to the use the prediction model (nomogram, calculator, etc.)	2 (7)
16. Model performance—report performance measures (with confidence intervals) for the prediction model	22 (73)
Section 5: Discussion	81 (90)
18. Limitations—Discuss any limitations of the study	30 (100)
19b. Interpretation—Give an overall interpretation of the results	30 (100)
20. Implications—Discuss the potential clinical use of the model and implications for future research	21 (70)
Section 6: Validation for Model type 2a, 2b, 3, and 4 (*N* = 13)	9 (17)
10c. Statistical analysis methods—describe how the predictions were calculated	0 (0)
10e. Statistical analysis methods—describe any model updating (recalibration), if done (*N* = 0)	0 (0)
12. Development versus validation—Identify any differences from the development data in setting, eligibility criteria, outcome, and predictors	5 (38)
13c. Participants (for validation)—show a comparison with the development data of the distribution of important variables	4 (31)
17. Model updating—report the results from any model updating, if done (*N* = 0)	0 (0)
19a. Interpretation (for validation)—discuss the results with reference to performance in the development data and any other validation data	0 (0)

*TRIPOD* Transparent Reporting of a Multivariable Prediction Model for Individual Prognosis or Diagnosis

**Fig. 4:**
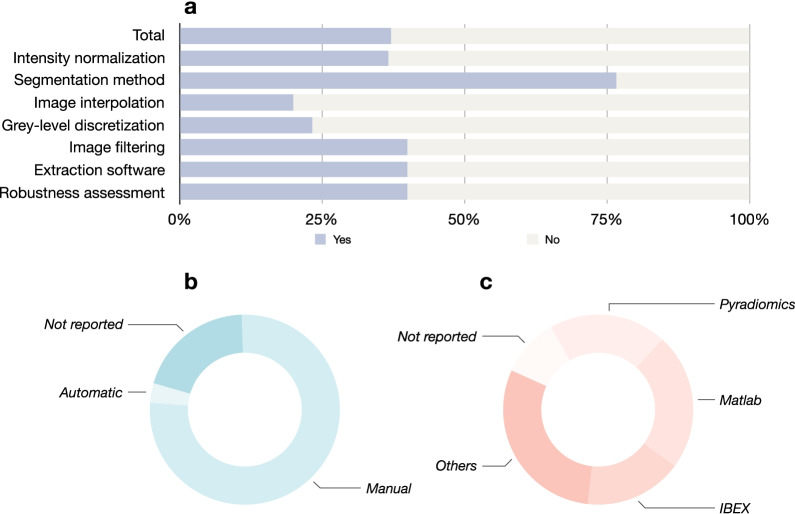
IBSI preprocessing steps performed in included studies. **a** Adherence rate of IBSI preprocessing steps; **b** segmentation method; **c** software for radiomics feature extraction. The other software included Omni-Kinetics, Artificial Intelligent Kit, AnalysisKit, Image J, FireVoxel, and MaZda

The overall adherence rate of IBSI preprocessing steps was 37.1% (78/210) (Fig. 4). The software for feature extraction varied among studies, including MATLAB (7/30), Pyradiomics (6/30), IBEX (5/30), and others. Three studies did not report the software used. Among these, Pyradiomics and IBEX were with IBSI compliance. The studies used manual (23/30) and automatic (1/30) methods for segmentation. However, one study did not report the segmentation method. The robustness assessment was performed in 40.0% (12/30) of the studies, all concerning the inter- and intra-reader agreements. Other preprocessing steps were sometimes conducted.

The results of the QUADAS-2 assessment are presented in Fig. 3. The risk of bias and application concerns relating to index testing were most frequently observed mainly due to the lack of external validation. The risk of bias in patient selection was rated as high in two studies due to the case–control design. Most of the studies did not provide the timing of scanning; therefore, the corresponding risk of bias was unclear. Individual assessment per study per element is present in Additional file 1: Tables S12 to S15.

### Meta-analysis

**Fig. 5:**
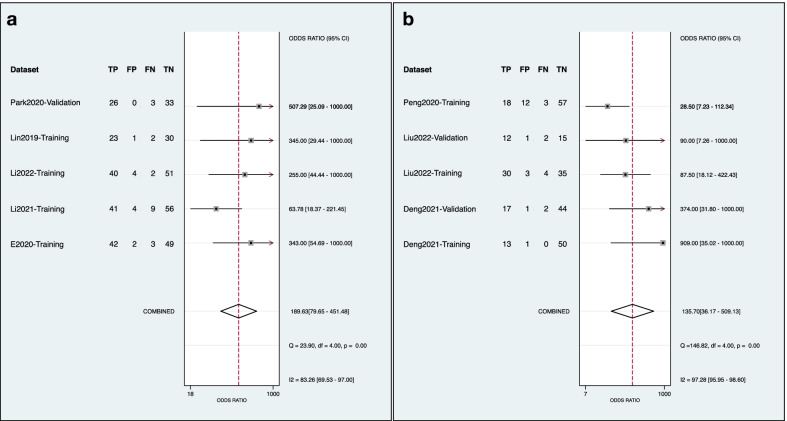
Forest plots of diagnostic odds ratio for differentiation diagnosis. **a** Autoimmune pancreatitis versus pancreatic cancer by CT; **b** mass-forming focal pancreatitis versus pancreatic cancer by MRI

The datasets for meta-analyses are present in Additional file 1: Table S16. The pooled analysis showed that the DOR (95% CI) of radiomics for distinguishing autoimmune pancreatitis versus pancreatic cancer by CT and mass-forming pancreatitis versus pancreatic cancer by MRI were 189.63 (79.65–451.48) and 135.70 (36.17–509.13), respectively (Fig. 5 and Table 4). However, their levels of evidence were both weak mainly due to the insufficient sample size. There was significant heterogeneity among studies, but the likelihood of publication bias was low. The trim and fill analysis demonstrated that there were missing datasets, but the adjusted diagnostic performance was still of statistical significance. The results of meta-analyses regardless of imaging modalities presented dramatic statistical significance (Additional file 1: Table S17). The corresponding plots of meta-analyses are present in Additional file 1: Figures S1 to S9.

**Table 4:** Diagnostic performance of meta-analyzed clinical questions

Clinical question	AIP versus PC by CT	MFP versus PC by MRI
Number of studies	6	4
Number of available datasets	5/8	5/6
Events/sample size	191/421	101/320
Pooled analysis		
DOR (95% CI)	189.63 (79.65–451.48)	135.70 (36.17–509.13)
*p* value for DOR	< 0.001	< 0.001
Sensitivity (95% CI)	0.90 (0.84–0.94)	0.90 (0.81–0.95)
Specificity (95% CI)	0.95 (0.92–0.97)	0.94 (0.86–0.98)
PLR (95% CI)	19.01 (10.51–34.40)	15.00 (5.94–37.92)
NLR (95% CI)	0.10 (0.06–0.17)	0.11 (0.06–0.56)
AUC (95% CI)	0.97 (0.95–0.98)	0.95 (0.93–0.96)
Heterogeneity		
Higgins I^2^ test (%)	83.26%	97.28%
Cochran’s Q test (*p* value)	< 0.01	< 0.01
Publication bias		
Egger’s test (*p* value)	0.060	0.050
Begg’s test (*p* value)	0.221	0.221
Deeks test (*p* value)	0.226	0.538
Trim and fill method		
Number of missing datasets	2	2
Adjusted DOR (95%CI)	135.11 (64.40–283.74)	53.89 (15.95–182.00)
Level of evidence	Weak	Weak

*AIP* autoimmune pancreatitis, *AUC* area under curve, *CI* confidential interval, *DOR* diagnostic odds ratio, *MFP* mass-forming pancreatitis, *NLR* negative likelihood ratio, *n/a* not applicable, *PC* pancreatic cancer, *PLR* positive likelihood ratio

### Correlations between study characteristics and quality

**Fig. 6:**
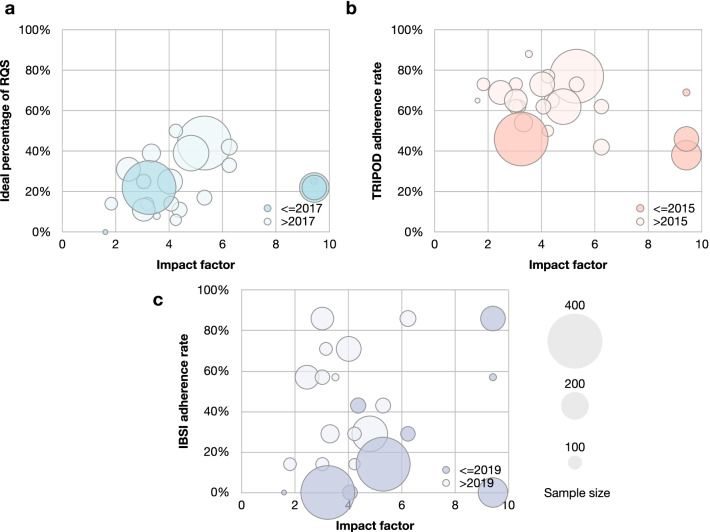
Correlations between study characteristics and quality. Swam plots of (**a**) ideal percentage of RQS, (**b**) TRIPOD adherence rate, and (**c**) IBSI adherence rate. The diameter of bubbles indicates the sample size of studies. Seven studies published on journals without impact factor were excluded. The lighter color indicates the studies after the publication of RQS, TRIPOD, and IBSI; the darker color indicates those before their publications

Figure 6 shows the potential correlation between study characteristics and its quality. The studies before and after the publication of the RQS, the TRIPOD checklist, or the IBSI guideline did not show obvious difference. Only the ideal percentage of RQS was considered to be related to the sample size (*r* = 0.456, *p* = 0.011). The results of subgroup analysis and correlation tests are present in Additional file 1: Tables S18 and S19. No difference of the ideal percentage of RQS, the TRIPOD adherence rate, and the IBSI adherence rate among subgroups has been found (all *p* > 0.05).

## Discussion

In our review, radiomics showed promising performance of diagnostic and prognostic models for multiple purposes in pancreatitis, but their levels of evidence were weak. The overall adherence rates of the RQS rating, the TRIPOD checklist, and the IBSI preprocessing steps were 38.3%, 61.3%, and 37.1%, respectively. The ideal percentage of RQS was positively related to the sample size. Our results implied that the level of evidence supporting clinical application and the overall study quality were suboptimal in pancreatitis radiomics research, requiring significant improvement.

Several reviews have summarized the use of radiomics in multiple pancreatic diseases from pancreatic cystic lesions to pancreatic tumors [15–22]. A comprehensive review reported that most of the pancreatic radiomics studies investigated focal pancreatic lesions, but only four studies discussed the pancreatitis [12]. In our review, radiomics has been most frequently applied to differential diagnosis of pancreatic cancer from autoimmune pancreatitis, chronic pancreatitis, or mass-forming pancreatitis. The misdiagnosis causes pancreatic cancer patients to miss the surgical opportunity, while the patients with inflammatory condition may receive unnecessary treatment. The accurate diagnosis of these lesions is hindered by mimicking imaging features [6]. Radiomics showed comparable and even better performance than radiologists’ assessment [38, 42, 46, 52, 56, 58], but their level of evidence supporting clinical translation is still weak. Therefore, more validation for the establishment of a sound evidence basis is the main issue for diagnostic. The prognosis prediction for acute pancreatitis is another topic of clinical significance. Although the CT severity index has been established for prognosis prediction of acute pancreatitis [4], the pancreatic parenchyma injury and extra-pancreatic inflammation are not visible enough in early pancreatitis. The conventional imaging features usually lag behind disease progression, which cannot help clinical decision-making. Current studies demonstrated the usefulness of radiomics in predicting severity, recurrence, progression, and extra-pancreatic necrosis [33, 35, 40, 41, 45, 59]. However, the studies were conducted by varying imaging modalities concerning separate outcomes, which do not allow further meta-analysis to establish any evidence. Besides, as a continuous disease progress, acute pancreatitis needs comprehensive prediction for multiple clinical outcomes. Corresponding models have not been developed yet. Thus, it is more urgent to encourage more investigation into prognosis.

The inadequate quality of radiomics studies has been addressed repeatedly [15–24, 27–29]. In accordance with previous reviews, several items were always lacking including test–retest analysis, phantom study, cutoff analysis, and cost-effectiveness analysis in RQS, the blinded method during outcome assessment, sample size calculation, and handling of missing data in TRIPOD, and details of image preprocessing in selected IBSI items. In spite of these common issues across radiomics studies, there are some non-oncology specific issues. Contrary to the oncological field, the concept of biological correlate did not clearly fit the current topic [17], since the inflammatory diseases do not always relate to genomics. In prognostic studies, comparison to “gold standard” is not suitable for non-oncological diseases without a widely accepted “gold standard,” while the tumor staging is usually employed as the “gold standard” of survival prediction. The TRIPOD items and IBSI preprocessing items were suitable for non-oncological studies, since they were not specified for oncological field. We found that the ideal percentage of RQS was positively related with the sample size. We suspected that the larger sample size might allow more sufficient validation, evaluation of calibration statics, and clinical utility assessment, which could gain a higher RQS rating.

Most of the radiomics studies were oncological, but radiomics has potential clinical application in the non-oncological field [30]. Several reviews have summarized the role of radiomics in non-oncological diseases, including mild cognitive impairment and Alzheimer’s disease [15], COVID-19 and viral pneumonia [16], and cardiac diseases [17]. The study quality evaluated by RQS was the main concern of these reviews. Their ideal percentage of RQS were 9.9%, 34.1%, and 19.4%, respectively. We suspected that the COVID-19 and viral pneumonia review reached a better RQS rating since the included studies were published recently with a relatively larger sample size, which allow adequate feature reduction and external validation. Actually, none of the studies in this review lacked the feature reduction, and all the studies performed validation [16]. In contrast, a significant number of previous studies did not perform feature reduction and validation. As a result, the other non-oncological radiomics reviews showed lower RQS ratings [15, 17]. Our review is in line with these non-oncological radiomics reviews with a comparable ideal percentage of RQS of 20.3%. Nevertheless, the feasibility of the TRIPOD checklist [28] and the IBSI preprocessing steps [29] have only been assessed in the oncological field. Our study initially tested and confirmed that they were useful in non-oncological field, but further validation is needed.

An evidence level rating tool has been tested in our review [31, 32]. The evidence level rating process is feasible to show the gap between academic research and clinical application in radiomics studies. It is necessary to employ this tool, since the dramatic model performance did not naturally guarantee a strong level of evidence supporting the clinical translation. However, this tool did not mention on which dataset a predictive model should be assessed, because this tool is originally developed for reviewing epidemic studies and clinical trials [31, 32]. It is recommended to perform the assessment of radiomics models on an external validation dataset [10, 11, 25]. We consider that future studies should determine the level of evidence based on results of meta-analyses of validation datasets.

We believed that the whole radiomics research community should participate in the improvement in methodological and reporting quality for a higher level of evidence to support the translation of radiomics. They need to get involved into this process, to critically appraise the study design, conduct and analyze the model, and report the study. Indeed, the IBSI guideline used in our review is an achievement gained by an independent international collaboration which works towards standardization of the radiomics methodology and reporting [11]. There are many other guidelines developed or under development by the radiomics and artificial intelligence community with the purpose to improve study quality, including Transparent Reporting of a multivariable prediction model for Individual Prognosis Or Diagnosis based on Artificial Intelligence (TRIPOD-AI) [63], Prediction model Risk Of Bias ASsessment Tool based on Artificial Intelligence (PROBAST-AI) [63], Quality Assessment of Diagnostic Accuracy Studies centered on Artificial Intelligence (QUADAS-AI) [64], Developmental and Exploratory Clinical Investigations of DEcision support systems driven by Artificial Intelligence (DECIDE-AI) [65], Standard Protocol Items: Recommendations for Interventional Trials–Artificial Intelligence (SPRIIT-AI) [66], Consolidated Standards of Reporting Trials–Artificial Intelligence (CONSORT-AI) [67], Standards for Reporting of Diagnostic Accuracy Study centered on Artificial Intelligence (STARD-AI) [68], Checklist for Artificial Intelligence in Medical Imaging (CLAIM) [69], etc. Their project teams and steering committees usually consisted of a broad range of experts to provide balanced and diverse views involving various stakeholder groups.

However, the importance of the participants varies with the stage from early scientific validation to later regulatory assessment. For offline preclinical validations, reporting guidelines and risk of bias assessment tools for radiomics model studies are used, emphasizing the methodological and reporting quality [63, 64]. During this stage, the researchers, authors, reviewers, and editors of radiomics studies play an important role to improve the methodological and reporting quality, and make sure only studies with adequate innovation are being published. Next, at the stage of safety and utility, the small-scale early live clinical evaluations are used to inform regulatory decisions and are part of the clinical evidence generation process [65]. With improvements of study quality, the radiomics research community could for the first time provide more robust scientific evidence for the translation of radiomics. Before clinical application, it is necessary to test the radiomics for safety and effectiveness in large-scale, comparative, and prospective trails [66–68]. Similar to the random clinical trials which are considered as the gold standard for drug therapies, the aim of these studies should be to provide stronger evidence for translation of radiomics from research application into a clinically relevant tool. Nevertheless, given the somewhat different focuses of scientific evaluation and regulatory assessment, as well as differences between regulatory jurisdictions, the health policy makers and legal experts may have a greater say in this stage.

The quality assessment results should be seen as a quality seal of the published results more than a way of underlining the possible weaknesses of the proposed model [70]. At present, the researchers are reticent in publishing the quality assessment results for their radiomics studies, and journals do not demand particular checklists for radiomics studies. Nevertheless, in this early stage of radiomics, the authors, editors, reviewers, and readers should be able to ascertain whether a radiomic study is compliant with good practice or whether the study has justified any noncompliance.

There are several limitations in our study. First, the RQS was far from perfect. Some of TRIPOD items may be not suitable for radiomics studies. We did not exhaust the IBSI checklist, but focused on preprocessing steps. Nevertheless, the current review served as an example for the application of these tools in the non-oncological field. Second, radiomics is considered as a subset of artificial intelligence, but we did not apply Checklist for Artificial Intelligence in Medical Imaging for our review [69]. This tool allows assessments on not only artificial intelligence in medical imaging that includes classification, image reconstruction, text analysis, and workflow optimization, but also general manuscript review criteria. However, many items in this tool are too general [71], and therefore hard to apply in radiomics. The tools we used could cover almost all the CLAIM items with more specific instructions. It would be interesting to assess the feasibility of CLAIM in radiomics, but it falls out of our study scope. Third, studies included in the current review focus on very different topics. It may not be fair to run meta-analyses of heterogenous studies, and this process gives insights into clinical questions with a limited number of studies [24, 72]. Indeed, only two selected clinical questions with similar settings were included in meta-analyses for evidence level rating. The increasing number of studies would allow more robust scientific data aggregation in the future. Still, this is a timely attempt to test the feasibility of the evidence level rating tool for radiomics.

In conclusion, more high-quality studies on prognosis of acute pancreatitis are encouraged, since it has great influence on clinical decision-making but could not be easily predicted by radiologists’ assessment. Although meta-analysis of studies showed fascinating potential in differentiating pancreatitis from pancreatic cancer, the level of evidence was weak. The current methodological and reporting quality of radiomics studies on pancreatitis is insufficient. Moreover, evidence rating is needed before radiomics can be translated into clinical practice.

## Supplementary Information

**Additional file 1: Supplementary Note S1.** Review Protocol. **Supplementary Note S2.** Search Strategy and Study Selection. **Supplementary Note S3.** Consensus Reached during Data extraction and Quality Assessment. **Supplementary Note S4.** Data Synthesis and Analysis Methods. **Supplementary Table S1.** Data Extraction Sheet. **Supplementary Table S2.** Methodological Quality according to RQS Checklist. **Supplementary Table S3.** Reporting Completeness according to TRIPOD Statement. **Supplementary Table S4.** Pre-processing Steps according to IBSI Guideline. **Supplementary Table S5.** Risk of Bias and Concern on Application Assessment according to QUADAS-2 Tool. **Supplementary Table S6.** Types of Prediction Model Studies Covered by The TRIPOD Statement. **Supplementary Table S7.** Trials Classifications for Image Mining Tools Development Process. **Supplementary Table S8.** Category of Five Levels of Supporting Evidence of Meta-analyzes. **Supplementary Table S9.** Study Characteristics of Included Studies. **Supplementary Table S10.** PICOT of Included Studies. **Supplementary Table S11.** Radiomics Methodological Consideration of Included Studies. **Supplementary Table S12.** RQS Rating per Study. **Supplementary Table S13.** TRIPOD Adherence per Study. **Supplementary Table S14.** Pre-processing Steps Performed in Each Study. **Supplementary Table S15.** QUADAS-2 Assessment per Study. **Supplementary Table S16.** Model Metrics of Studies Included in Meta-analysis. **Supplementary Table S17.** Diagnostic performance of meta-analyzed clinical questions regardless of imaging modality. **Supplementary Table S18.** Subgroup Analysis of Study Quality according to Study Characteristics. **Supplementary Table S19.** Correlation between Ideal Percentage of RQS, TRIPOD Adherence Rate, Sample Size and Impact Factor. **Supplementary Figure S1.** Forrest Plot of Diagnostic Odds Radio. **Supplementary Figure S2.** Forrest Plot of Pooled Sensitivity. **Supplementary Figure S3.** Forrest Plot of Pooled Specificity. **Supplementary Figure S4.** Forrest Plot of Pooled Positive Likelihood Ratio. **Supplementary Figure S5.** Forrest Plot of Pooled Negative Likelihood Ratio. **Supplementary Figure S6.** HSROC Curve of the Model Performance. **Supplementary Figure S7.** Funnel plot of Studies Included in Meta-analysis. **Supplementary Figure S8.** Deeks Funnel Plot of Studies Included in Meta-analysis. **Supplementary Figure S9.** Trim and Fill Analysis of Studies Included in Meta-analysis.

**Additional file 2:** PRISMA checklist.

## Data Availability

All data generated or analyzed during this study are included in this published article and its Additional file 1.

## Abbreviations

CI: Confidence interval
DOR: Diagnostic odds ratio
HSROC: Hierarchical summary receiver operating characteristic
IBSI: Image Biomarker Standardization Initiative
QUADAS-2: Modified Quality Assessment of Diagnostic Accuracy Studies
RQS: Radiomics Quality Score
TRIPOD: Transparent Reporting of a Multivariable Prediction Model for Individual Prognosis or Diagnosis
