# Supplementary material for: A systematic review of radiomics in pancreatitis: applying the evidence level rating tool for promoting clinical transferability
Source: Insights Imaging. 2022 Aug 20;13:139. doi: 10.1186/s13244-022-01279-4 (PMC9391628; doi:10.1186/s13244-022-01279-4)

ELECTRONIC SUPPLEMENTARY MATERIAL

A systematic review of radiomics in pancreatitis: applying the evidence level rating tool for promoting clinical transferability

List of Supplementary Materials

Supplementary Note S1 Review Protocol
Supplementary Note S2 Search Strategy and Study Selection
Supplementary Note S3 Consensus Reached during Data extraction and Quality Assessment
Supplementary Note S4 Data Synthesis and Analysis Methods

Supplementary Table S1 Data Extraction Sheet
Supplementary Table S2 Methodological Quality according to RQS Checklist
Supplementary Table S3 Reporting Completeness according to TRIPOD Statement
Supplementary Table S4 Pre-processing Steps according to IBSI Guideline
Supplementary Table S5 Risk of Bias and Concern on Application Assessment according to QUADAS-2 Tool
Supplementary Table S6 Types of Prediction Model Studies Covered by The TRIPOD Statement
Supplementary Table S7 Trials Classifications for Image Mining Tools Development Process
Supplementary Table S8 Category of Five Levels of Supporting Evidence of Meta-analyzes

Supplementary Table S9 Study Characteristics of Included Studies
Supplementary Table S10 PICOT of Included Studies
Supplementary Table S11 Radiomics Methodological Consideration of Included Studies
Supplementary Table S12 RQS Rating per Study
Supplementary Table S13 TRIPOD Adherence per Study
Supplementary Table S14 Pre-processing Steps Performed in Each Study
Supplementary Table S15 QUADAS-2 Assessment per Study
Supplementary Table S16 Model Metrics of Studies Included in Meta-analysis
Supplementary Table S17 Diagnostic performance of meta-analyzed clinical questions regardless of imaging modality
Supplementary Table S18 Subgroup Analysis of Study Quality according to Study Characteristics
Supplementary Table S19 Correlation between Ideal Percentage of RQS, TRIPOD Adherence Rate, Sample Size and Impact Factor

Supplementary Figure S1 Forrest Plot of Diagnostic Odds Radio
Supplementary Figure S2 Forrest Plot of Pooled Sensitivity
Supplementary Figure S3 Forrest Plot of Pooled Specificity
Supplementary Figure S4 Forrest Plot of Pooled Positive Likelihood Ratio
Supplementary Figure S5 Forrest Plot of Pooled Negative Likelihood Ratio
Supplementary Figure S6 HSROC Curve of the Model Performance
Supplementary Figure S7 Funnel plot of Studies Included in Meta-analysis
Supplementary Figure S8 Deeks Funnel Plot of Studies Included in Meta-analysis
Supplementary Figure S9 Trim and Fill Analysis of Studies Included in Meta-analysis

Supplementary Note S1 Review Protocol

PROSPERO registration number: Not available now
PROSPERO registration ID: 311114

Review question

This systematic review assesses the study quality and clinical value of the literature published on radiomics, texture analysis or histogram analysis of pancreatitis for diagnosis, prediction or prognosis.

Searches

Primary publications concerning radiomics, image texture analysis or histogram analysis in patients with pancreatitis will be included in this review. Electronic databases including PubMed, EMBASE, Web of Science, China National Knowledge Infrastructure, and Wanfang Data will be searched. Literature search strategies will be developed using medical subject headings (MeSH) and derived words, including radiomics, textural analysis, histogram, pancreatitis, etc. There are studies using quantitative image analysis technique before the term radiomics has been highlighted by Limbin P in 2012; therefore, no restriction will be made regarding publication period. The search strategy will include only terms relating to the review question. Publications must be available in full text in English, Chinese, Japanese, German or French.

Condition or domain being studied

Pancreatitis is an inflammatory disorder of the pancreas, including acute pancreatitis and chronic pancreatitis. Imaging is important in diagnosis and prognosis in pancreatitis. However, it is sometimes hard to differentiate chronic pancreatitis from some malignant tumors by imaging, and it is needed to predict the prognosis of acute pancreatitis to guide the clinical treatments. Radiomics, a brunch of image mining methods, currently provides the opportunity for radiologists to improve the diagnosis, prediction or prognosis performance in pancreatitis. There is no systematic review concerning this topic; therefore, we will assess the study quality and clinical value of the literature published on radiomics, texture analysis or histogram analysis of pancreatitis for diagnosis, prediction or prognosis.

Participants/population

Participants' inclusion criteria:
1) patients with pancreatitis;
2) patients had undergone at least one pre- treatment pre- or post-treatment imaging;
3) a quantitative image analysis for diagnosis, prediction or prognosis of patients was established.

Participants' exclusion criteria:
1) not human patients, e. g. cell line, animal experiments;
2) not pancreatitis, e. g. prognosis of pancreatic cancer;
3) no performed imaging procedure, e. g. studies based on only clinical datasets;
4) no quantitative image analysis performed or model established.

Intervention(s), exposure(s)

Patients with pancreatitis underwent at least one pre- treatment pre- or post-treatment imaging with a quantitative image analysis performed or model established based on these imaging data.

Comparator(s)/control

Standard-of-care imaging.

Types of study to be included

This systematic review will include primary research assessing the role of quantitative image analysis performed or model established in patients with pancreatitis. Studies may be prospective or retrospective and randomized or non-randomized and shall investigate the use of texture analysis for diagnostic, prognostic or predictive purposes on cross-sectional imaging of human patients with pancreatitis. The reference lists of included studies and relevant reviews were screened for additional potentially eligible articles. However, reviews, technical reports, letters to editors, comments to published studies, conference proceedings, case reports and brief communications, and articles with insufficient information for assessing the methodological quality will be excluded.

Main outcome(s)

The characteristics of included study will be summarized. The methodological quality, reporting quality, image

pre-processing steps, and risk of bias and concern on application, will be assessed.

Measures of effect
The eligible articles were assessed according to Radiomics Quality Score (RQS) checklist for methodological quality, the Transparent Reporting of a multivariable prediction model for Individual Prognosis Or Diagnosis (TRIPOD) statement for reporting quality, the Image Biomarkers Standardization Initiative (IBSI) guideline for image pre-processing steps, and the modified Quality Assessment of Diagnostic Accuracy Studies (QUADAS-2) tool for risk of bias and concern on application.

Additional outcome(s)
If a sufficient number of studies attempts to answer a similar question, a meta-analysis may be performed to present the performance of those radiomics models.

Measures of effect
Measures will be made during the data analysis phase.

Data extraction (selection and coding)
Study inclusion criteria:
1) studies are reported in English, Chinese, Japanese, German or French with institutional full-text availability;
2) the cohort consists of patients with pancreatitis;
3) patients had undergone at least one pre- or post-treatment imaging;
4) a quantitative image analysis or model was established.

Study exclusion criteria:
1) duplicate studies;
2) reviews, technical reports, letters to editors, comments to published studies, conference proceedings, case reports, brief communications and articles with insufficient information for assessing the study quality;
3) studies are reported other than English, Chinese, Japanese, German or French;
4) not human, not quantitative image analysis studies.

A data collection tool will be established based on similar reviews and then trialed on two randomly chosen studies, which fulfilled all the inclusion criteria. These shall be used to train reviewers to appropriately apply the data extraction tool.

Risk of bias (quality) assessment
The eligible articles were assessed according to Radiomics Quality Score (RQS) checklist for methodological quality, the Transparent Reporting of a multivariable prediction model for Individual Prognosis Or Diagnosis (TRIPOD) statement for reporting quality, the Image Biomarkers Standardization Initiative (IBSI) guideline for image pre-processing steps, and the modified Quality Assessment of Diagnostic Accuracy Studies (QUADAS-2) tool for risk of bias and concern on application.

Strategy for data synthesis
A narrative synthesis will be provided with information presented in the text and/or tables to summarize and explain the characteristics and findings of the included studies. A quantitative synthesis will be done if the included studies are sufficiently homogenous. All analysis will be based on aggregate data.

Analysis of subgroups or subsets
If a sufficient number of studies are identified, subgroup analyses may be performed. If a sufficiently homogeneous subset of studies analyzed a single outcome parameter, a meta-analysis of this subgroup may be attempted.

Type and method of review
Diagnostic; Prognostic; Systematic review; Digestive system

Conflicts of interest
The authors declare that they have no competing interests.

Supplementary Note S2 Search Strategy and Study Selection

1. Study Search Strategy

1. PubMed Search Strategy

Available via https://pubmed.ncbi.nlm.nih.gov
Preliminary search date: 15 Feb 2022
Articles retrieved: 122
Formal search date: 28 Feb 2022
Articles retrieved: 122

("pancreatitis" OR "pancreatitis"[Mesh]) AND (radiomic* OR textur* OR histogram*)

1.2 Embase Search Strategy

Available via www.embase.com
Preliminary search date: 15 Feb 2022
Articles retrieved: 221
Formal search date: 28 Feb 2022
Articles retrieved: 221

('pancreatitis'/exp OR 'pancreatitis':ti,ab,kw) AND ('radiomic':ti,ab,kw OR 'radiomics'/exp OR 'radiomics':ti,ab,kw OR 'textural':ti,ab,kw OR 'texture'/exp OR 'texture':ti,ab,kw OR 'histogram'/exp OR 'histogram':ti,ab,kw)

1.3 Web of Science Search Strategy

Available via apps.webofknowledge.com
Preliminary search date: 15 Feb 2022
Articles retrieved: 186
Formal search date: 28 Feb 2022
Articles retrieved: 186

(TS=(pancreatitis)) AND (TS=(radiomic*) OR TS=(radiomics*) OR TS=(textural*) OR TS=(texture*) OR TS=(histogram*))

1.4 China National Knowledge Infrastructure Search Strategy

Available via http://www.cnki.net
Preliminary search date: 22 Feb 2022
Articles retrieved: 23
Formal search date: 28 Feb 2022
Articles retrieved: 23

"胰腺炎" * ("影像组学" + "直方图" + "纹理")
English translation: pancreatitis * (radiomics + histogram + texture)

1.5 Wanfang Data Search Strategy

Available via https://www.wanfangdata.com.cn
Preliminary search date: 22 Feb 2022
Articles retrieved: 35
Formal search date: 28 Feb 2022
Articles retrieved: 35

"胰腺炎" AND ("影像组学" OR "直方图" OR "纹理")
English translation: pancreatitis AND (radiomics OR histogram OR texture)

2. Study Selection

2.1 Studies included in systematic review

Study inclusion criteria:

1) studies are reported in English, Chinese, Japanese, German or French with institutional full-text availability;

2) the cohort consists of patients with pancreatitis;
3) patients had undergone at least one pre- or post-treatment imaging;
4) a quantitative image analysis or model was established.

Study exclusion criteria:
1) duplicate studies;
2) reviews, technical reports, letters to editors, comments to published studies, conference proceedings, case reports, brief communications and articles with insufficient information for assessing the study quality;
3) studies are reported other than English, Chinese, Japanese, German or French;
4) not human, not quantitative image analysis studies.

Contact with the authors was sought if the full-text version was not accessible otherwise. The reference lists of included studies and relevant reviews identified through the search were screened for additional, potentially eligible articles. Two reviewers both with 4-year-experience in radiology and radiomics research screened and selected studies independently. One of these two reviewers can read articles in English, Chinese, Japanese, German and French. In case of disagreements, a third reviewer with 30-year-experience in abdominal radiology would be consulted.

2.2 Studies included in meta-analyzes
As predetermined in the review protocol, if a sufficient number of studies attempts to answer a similar question, a meta-analysis could be performed. In current study, differential diagnosis of autoimmune pancreatitis vs. pancreatic cancer by CT and PET, and mass-forming pancreatitis vs. pancreatic cancer by CT and MRI, were repeatedly addressed. Therefore, these studies were included in the meta-analysis.

The studies included in meta-analysis should meet following criteria:
1) studies used a radiomic model to answer one of the above three clinical questions;
2) studies with clearly defined criteria for histological assessment of grading of chondrosarcomas;
3) studies with documented sensitivity (Se), specificity (Sp), accuracy, positive predictive value (PPV), negative predictive value (NPV) and likelihood ratio (LR), diagnostic odds ratio (DOR), or with those could be calculated using published data.

The studies were excluded from meta-analysis because:
1) overlapping datasets;
2) Two-by-two tables not documented, and could be calculated using published data.

If multiple radiomic models were reported in a study, only the one with the best discrimination performance was included. If multiple radiomic models were aimed to answer different clinical questions, all of them were included. The two-by-two tables were directly extracted, if documented, or reconstructed based on available data, by one reviewer and then checked by another. In case of disagreements, a third reviewer with 30-year-experience in abdominal radiology would be consulted. One of the reviewers has significant statistical expertise, and performed the meta-analysis.

Supplementary Note S3 Consensus Reached during Data extraction and Quality Assessment

1. Methodological Quality according to RQS Checklist

The RQS consists of 16 items concerning crucial aspects of radiomics studies, to assess their methodological quality. The reviewers performed RQS evaluation according to six key domains as previous reported. Two reviewers who both with 4-year-experience in radiology and radiomics research discussed with a third reviewer with 30-year-experience in abdominal radiology, and made a consensus on additional topics of RQS considering the characteristics of current review.

The following topics reached a consensus: (1) Multiple segmentation (domain 1): when there were two or more readers, the article earned an additional point if segmentation variability was considered. Automatic segmentation using a convolutional neural network or other automatic software earned a point as the method pursued better segmentation reproducibility. (2) Validation (domain 2): if cross-validation or nested cross-validation was performed only within the training set, it was considered missing validation and scored -5 points, as previously described. If validation was performed on a dataset from the same institution, it scored +2 points. If the validation was based on a dataset from another institute, it scored +3 points. (3) Comparison with the gold standard (domain 3): in studies that aimed to differentiate subtypes of pancreatitis and pancreatic cancer, the judgment of radiologists before the post-operation histological assessment was considered the gold standard. Therefore, studies comparing the diagnostic performance of radiomics with that of radiologists scored 1 point. As there are several scoring systems for the prediction of prognosis in patients with pancreatitis, such as Acute Physiology and Chronic Health Evaluation (APACHE) II, the bedside index for severity in acute pancreatitis (BISAP), and CT severity index (CTSI), if the studies comapered radiomics models with these scoring systems, the studies scored 1 points on this topic. (4) Biologic correlation (domain 3): studies that attempted to elucidate the possible correlations between radiomic features and microenvironments of lesions (e. g., variance measures the deviation of gray levels from the mean and represents the extent of the histogram, which may reflect on morphologic imaging performance) scored 1 point. We did not employ the criteria that correlations between radiomic features and genetic mutation status, since genetic mutation detection has not been widely accepted in the clinical settings. (5) Clinical utility (domain 3): clinical utility is thought to be achieved when a biomarker leads to net improvement of health outcomes or provides information useful for prevention, diagnosis, treatment, and management of a disease. A study earned 2 points if the clinical utility was objectively 'measured', such as decision curve analysis to demonstrate net improvement. On the other hand, discussion of the potential utility of radiomics without proper analysis did not earn additional points. (6) Open science and data (domain 6): the study gains 1 point for open science, if similar statements were made: "The model, its implementation instructions, all required files for data extraction and processing are available in the online study repository."

2. Reporting Completeness according to TRIPOD Statement

The TRIPOD checklist, consisting of 37 items in 22 criteria, was applied to determine the reporting completeness of the included prediction models. Since the TRIPOD checklist was originally produced for the clinical prediction model, it was partially modified for application in radiomics studies. Two items relevant to supplementary materials and funding (items 21,22) were excluded as previous suggested. Two reviewers who both with 4-year-experience in radiology and radiomics research discussed with a third reviewer with 30-year-experience in abdominal radiology, and made a consensus on additional topics of TRIPOD considering the characteristics of current review.

The following topics reached a consensus: (1) Title (item 1): considered as complete if all elements of the type of study (development/validation/incremental value/or combination), the target population, and outcome are included. (2) Study objective (items 2 and 3b): considered as complete if 'development' and/or 'validation' is explicitly written. Synonyms instead of development such as 'establish', 'build', 'investigate', and 'evaluate' were not considered as complete. (3) Source of data (item 4a and 4b): whether the study was conducted in a randomized controlled trial, cohort, or registry with a consecutive, random, or convenience series. A study was considered as complete for item 4a when the terms 'retrospective' or 'prospective', inclusion period, and inclusion center were mentioned. A study was considered as complete for item 4b when the name of open-source data was provided, or declaim that the study was performed based on institutional dataset with a specific inclusion period. (4) Clearly define all predictors used, including how and when they were measured (item 7a): the radiomics studies involve quantitative feature extraction through an automated process; thus, the element 'when' was ignored. Report any actions to blind assessment of predictors for the outcome and

other predictors (item 7b): if radiomics studies were based on regions-of-interest and the blindness of readers to the reference standard was considered, they were recorded as complete. If ‘blind’ or ‘unaware of’ the reference standard was not explicitly written, it was considered as incomplete. Automatic segmentation was considered as complete. (5) Specify type of the model, all procedures, and methods for internal validation (item 10b): considered as complete if all three elements, model type (e.g., logistic regression, Cox proportional hazards model), feature selection procedure to control overfitting, and methods of internal validation (cross-validation, bootstrap sample), were included. A regularization or penalization method such as the least absolute shrinkage and selection operator (LASSO) was considered as both a feature selection procedure and internal validation, as it contains 10-fold cross-validation as a default setting. (6) Specify measure of model performance (item 10d): the article was considered as complete if both the discrimination and calibration index were written. (7) Flow of participants (item 13a): considered as complete for 13a if a diagram or text description with the numbers of screened patients, excluded patients and included patients was provided. (8) Describe how the predictions were calculated (item 10c), present the full prediction models (item 15a), and explain how to use the prediction model (item 15b): these items determine if an article describes how the obtained model predicted the outcome probabilities for an individual. If the articles described this in the methods (item 10c) and contained a full prediction model including all regression coefficients and the intercept or baseline hazard for a particular time point, they were considered as complete for item 15a. If the study contained explicit formula or a nomogram, the study was considered as complete for item 15b. (9) Model update (items 10e and 17): If an article describes methods to adjust (recalibrate) or update a previously developed prediction model, the article is scored. This is different from ‘comparison with gold standard’ in RQS criterion 13, in that it requires recalibration of regression coefficients and hazard ratios in the pre-existing model, and was scored if it was completely reported. (10) Difference between development and validation cohort: (items 12 and 13c): considered as complete if a table comparing developing and testing dataset or text description was provided.

3. Pre-processing Steps according to IBSI Guideline

The IBSI guideline provides a comprehensive reporting checklist in the for radiomics studies. Since most of the items in IBSI checklist overlapped with the RQS or TRIPOD checklists, we only included following seven items relevant to pre-processing steps: (1) intensity normalization, (2) segmentation method, (3) image interpolation/iso-voxel resampling, (4) grey-level discretization, (5) image filtering, (6) IBSI compliance of radiomics extraction software, (7) robustness of imaging biomarkers assessment. Two items (bias-field correction, skull stripping) which had been assessed in a previous study were excluded, because they were specific to brain MRI researches. Two reviewers who both with 4-year-experience in radiology and radiomics research discussed with a third reviewer with 30-year-experience in abdominal radiology, and made a consensus on additional topics of IBSI pre-processing steps considering the characteristics of current review.

The following topics were discussed to achieve consensus: (1) Image filtering: We assessed “yes” for this item when study clearly demonstrated that they extracted radiomics feature from filtered images, or a term “wavelet feature”, “filtered feature” or “Laplacian of Gaussian feature” was mentioned. (2) Robustness of imaging biomarkers assessment: We assessed “yes” for this item when the study performed a phantom study for repeatability or reproducibility, or the study measured the inter-observer agreement between multiple observer who performed the segmentation. We expanded the definition of robustness assessment of imaging biomarkers because it is not always suitable to perform a phantom study.

4. Risk of Bias and Concern on Application Assessment according to QUADAS-2 Tool

The QUADAS-2 tool was developed for the risk of bias and concern of application assessment. The tool was tailored to our study by two reviewers who both with 4-year-experience in radiology and radiomics research through modifying signaling questions specific to current study. The disagreements were resolved by discussion with a third reviewer with 30-year-experience in abdominal radiology, and made a consensus on additional topics of QuADAS-2 tool considering the characteristics of current review.

The following topics were discussed to achieve consensus: (1) Patient selection: we used the three original signal questions for our review: “was a consecutive or random sample of patients enrolled?”, “was a case–control design avoided?”, and “did the study avoid inappropriate exclusions?”, since these questions were suitable for radiomics studies. The studies which avoided case-control design, and clearly declare consecutive or random sample inclusion were rated as “low risk”. We considered the studies that have provided a clear inclusion period as consecutive. The studies with case-control design or inappropriate

exclusions, were rated as “high risk”. The studies without a clear declaration of consecutive or random sample inclusion were rated as “unclear” (2) Index test: the original signal questions of this domain were “were the index test results interpreted without knowledge of the results of the reference standard?” and “if a threshold was used, was it prespecified?” However, the radiomics process is an automatic pipeline, and the knowledge of the results of the reference standard would has limited influence on the results interpretation, since the researchers who did the segmentation did not directly make the interpretation. For radiomics studies, it is not possible to prespecify a threshold for radiomics features. Therefore, these two questions were not considered. For replacement, we have added three additional questions specified for radiomics methodology: “were the imaging acquisition protocol, image processing approach described in detail?”, “were the segmentation method(s), and feature extraction software described in detail?”, and “was the validation independent (i. e. external)?”. We considered cross-validation and bootstrapping as internal validation. The external validation may be performed using three different strategies including temporal (i.e., data obtained in newly recruited patients), geographic (i.e., data collected in a different institution), or split-sample (i.e., data split from the entire dataset and kept untouched for the test). We considered these three modified signal questions were tightly related to the risk of bias of radiomics workflow. The studies without detailed imaging acquisition protocol, image processing approach, the segmentation method(s), and feature extraction software, or validated with an internal dataset were rated as “high risk”. The studies with detailed methodological description and external validation were rated as “low risk”. (3) Reference standard: we used one of the two original signal questions for our review: “is the reference standard likely to correctly classify the target condition?”. We considered pathohistological assessment as the adequate standard for malignant tumors in diagnostic researches. Both excision or biopsy were acceptable sample source for pathohistological assessment. For diagnosis of benign diseases, pathohistological assessment was considered as adequate, and clinical diagnosis was also acceptable. For prognosis researches, an adequate follow-up with suitable examinations were needed. We did not used the other original signal question: “were the reference standard results interpreted without knowledge of the results of the index test?”, since the results interpretation by reference standard was always before the results interpretation by the radiomics model, and these two processes were performed separately. The studies with adequate standard were rated as “low risk” and those using suboptimal standard were rated as “high risk”. (4) Follow and timing: there were four original questions: “was there an appropriate interval between index tests and reference standard?”, “did all patients receive a reference standard?”, “did all patients receive the same reference standard?”, and “were all patients included in the analysis?”. We only used the first signal question. Since the timing of radiomics workflow did not influence the results, we focus on the timing of imaging and reference standard, especially the interval between imaging and surgery or biopsy. We did not use the second and third signal question, because all the patients had had to be assessed with adequate standard before the radiomics workflow began. We did not use the last signal question, because not all the patients were included in the analysis due the nature of radiomics workflow, which always divided patients into training dataset and validation dataset. The modified signal question was “was there an appropriate interval between imaging and reference standard?”. If the reference standard was pathohistological assessment after surgery or biopsy, the studies with a clear declaration of adequate interval between imaging and surgery or biopsy were rated as “low risk”; those without a clear declaration were rated as “unclear”; those with a clear declaration of inadequate interval were rated as “high risk”. If the reference standard was clinical diagnosis, or follow-up for prognosis purpose, the studies were rated as “low risk”, since there was not suitable to set an appropriate interval.

Supplementary Note S4 Data Synthesis and Analysis Methods

1. Statistical Analysis

The SPSS software version 26.0 was used for statistical analysis. A two-tailed *p*-value < 0.05 was recognized as statistical significance, unless otherwise specified. In case a score of at least one point for each item was obtained without minus points, it was considered to have basic adherence, as those which have been reported. 16 items of the RQS were scored. The RQS score and percentage of the ideal score were described as score and percentage of score to ideal score for each item, respectively. A total of 35 items on the TRIPOD checklist was scored. During the calculation of TRIPOD, the "if done" or "if relevant" items (5c, 11, and 14b) and validation items (10c, 10e, 12, 13, 17, and 19a) were excluded from both the denominator and numerator. 7 items from the IBSI guidelines are scored. The basic adherence of items in TRIPOD checklist and IBSI guideline was defined as those have been reported. The adherence rate of RQS rating, TRIPOD checklist and IBSI guideline were calculated as proportion of the number of articles with basic adherence to number of total articles. The result of QUADAS-2 risk of bias and application concern assessment was summarized as proportions of high risk, low risk and unclear.

Subgroup analysis was performed to determine whether factor influenced on the study quality including journal type, first authorship, biomarker, and imaging modality. According to normality test results, independent *t*-test or Mann-Whitney's U-test were used for intergroup differences, and one way analysis of variance or Kruskal-Wallis H-test were applied for multiple comparisons. The Bonferroni method was used for post-hoc correction (p = 0.05/6 = 0.008). The Pearson or Spearman correlation test was used for the correlation analysis between RQS, TRIPOD, sample size and impact factor.

2. Meta-analysis

The Stata software version 15.1 with metan, midas, and metandi packages was employed for meta-analysis. In current review, the value of radiomics in differential diagnosis of autoimmune pancreatitis vs. pancreatic cancer by CT and PET, and mass-forming pancreatitis vs. pancreatic cancer by MRI, were repeatedly addressed. Therefore, these clinical questions were included in the meta-analysis.

If multiple radiomic models were reported in a study, only the one with the highest area under the receiver operating curve (AUC), or the highest Youden' s index or the highest accuracy, if no AUC was reported, was included. If multiple radiomic models were aimed to answer different clinical questions, all of them were included. Due to the relatively insufficient sample size of included studies, we conducted the meta-analyzes with all available data.

One reviewer directly extracted or reconstructed the two-by-two tables based on available data; and then another reviewer cross-checked the results. The diagnostic odds ratio (DOR) and its corresponding 95% confidence interval (CI) were quantitatively synthesized as the main effect using random-effect model, and the corresponding *p*-value was calculated. Sensitivity, specificity, positive and negative likelihood ratio and their 95%CIs were also calculated, and relevant forest plots were obtained. Additionally, forest plots were drawn to show the heterogeneity in sensitivity, specificity, positive likelihood ratio, negative positive likelihood ratio and diagnostic odds ratio. A hierarchical summary receiver operating characteristic (HSROC) curve was plotted to visually show the diagnostic accuracy.

For assessment of heterogeneity between the included studies, the Cochran's Q and the I^2 statistic were calculated. Measuring inter-study dispersion assumes that, if all studies were methodologically identical and variation in results were only due to the random selection of study participants, the effect sizes would follow a chi-squared distribution. Cochran's Q assesses the hypothesis that the distribution of results is homogenous and p-values < 0.05 would generally lead to the rejection of this null-hypothesis. As with a small number of studies Cochran's Q can be distorted, I^2, a measure for how much of the variability between effect size estimates is due to methodological heterogeneity rather than sampling error, was also reported. I^2 values of 25% and less are usually considered to be low or unimportant, 25% to 50% moderate and values above 75% are considered high. Difference between the 95% confidence region and prediction region in the HSROC curve was used to visually assess the heterogeneity, and a large difference indicate heterogeneity.

A funnel plot and Deeks funnel plot were drawn to visually assessed publication bias. Egger's and Begg's tests were performed to assess the publication bias and a p-value > 0.1 indicated a low publication bias. Trim

and fill method was used to estimate the number of missing studies. A Deeks funnel asymmetry test was also constructed to explore the risk of publication bias, and a p-value > 0.10 indicated a low publication bias. Excess significance evaluation and 10% credibility ceiling calculation were not necessary in current review. Since none of the clinical question included in meta-analysis met the criteria of >1000 samples.

The code used for Stata programming is present as follows.

```
// tp = true positive, fp = false positive, fn = false negative, tn = ture nagative
// For sensitivity, specificity, positive likelihood ratio, negative likelihood ratio and diagnostic odds ratio plot
midas tp fp fn tn, res(all)
midas tp fp fn tn, uforest(dss) id (studyid) ford fors
midas tp fp fn tn, uforest(dlr) id (studyid) ford fors
midas tp fp fn tn, texts(0.6) uforest(dlor) id (studyid) ford fors
// For HSROC curve plot
metandi tp fp fn tn, plot
midas tp fp fn tn, sroc(conf)
// For heterogeneity
midas tp fp fn tn, res(het)
// For funnel plot, and Egger's and Begg's test
gen d=sqrt(3) * (log(tp)+log(tn)-log(fp)-log(fn))/3.14
replace d=sqrt(3) * (log(tp+0.5)+log(tn+0.5)-log(fp+0.5)-log(fn+0.5))/3.14 if d==.
gen vard=3 * (1/tp+1/fp+1/fn+1/tn)/(3.14 * 3.14)
replace vard=3 * (1/(tp+0.5)+1/(fp+0.5)+1/(fn+0.5)+1/(tn+0.5))/(3.14 * 3.14) if vard==.
gen sed=sqrt(vard)
metafunnel d sed
metabias d sed, egger
metabias d sed, begg
// For Deeks funnel plot, and Deeks funnel plot asymmetry test
midas tp fp fn tn, pubbias
// For trim and fill method analysis
gen logor= log((tp * tn)/(fp * fn))
replace logor=log(((tp+0.5) * (tn+0.5))/((fp+0.5) * (fn+0.5))) if logor==.
gen selogor=sqrt(1/tp+1/fp+1/fn+1/tn)
replace selogor=sqrt(1/(tp+0.5)+1/(fp+0.5)+1/(fn+0.5)+1/(tn+0.5)) if selogor==.
metatrim logor selogor, eform funnel
```

3. Clinical value and Level of Evidence

To assess the gap from clinical application, the model type and the phase of image mining studies were determined according to TRIPOD statement (type 1a, type 1b, type 2a, type 2b, type 3, and type 4), and a previous review (discovery science, phase 0, phase I, phase II, phase II, and phase IV). The pieces of evidence supporting clinical values of radiomics models were categorized into five levels (convincing, highly suggestive, suggestive, weak, and not suggestive) based on results of meta-analysis.

The process of evidence category requires multiple results based on meta-analysis, including a p value of pooled analysis with random model, events calculation, the largest study reaches statistical significance, assessment of heterogeneity by I^2 assessment, the null value excluded by the 95% predictive interval, small-study effects by Egger's test, excess significance, and 10% credibility ceiling calculation.

Supplementary Table S1 Data Extraction Sheet

Field	Item
Bibliographical Information	The Title of The Study
	The First Authorship of The Study
	Published Year
	Published Journal
	Impact Factor of Published Journal
	Published Volume
	Published Issue
	Published Page
	Country
	Study ID, determined by First Author + Year, + Journal if needed
Study Characteristics	Study Design
	Patient Condition
	Patient Gender
	Patient Age
	Imaging Modality
	Predictor
	Outcome
	Reference Standard
	Data Splitting
Radiomics Considerations	ROI Segmentation
	Radiomics Feature Extraction Details
	Radiomics Feature Reduction Details
	Radiomics Feature Selection Details
	Selector
Model Metrics	Sample Size
	Number of Events (True Positive, False Positive, False Negative, True Negative)
	Sensitivity
	Specificity
	Accuracy
	Positive Predictive Value (PPV)
	Negative Predictive Value (NPV)
	Positive Likelihood Ratio (PLR)
	Negative Likelihood Ratio (NLR)
	Diagnostic Odds Ratio (DOR)

Note: none.

Supplementary Table S2 Methodological Quality according to RQS Checklist

Domain	RQS#	RQS scoring item	Points and Interpretation
**Domain 1:** Protocol quality and stability in image and segmentation (0 to 5)	1	**Image protocol quality** - well-documented image protocols (for example, contrast, slice thickness, energy, etc.) and/or usage of public image protocols allow reproducibility/replicability	+ 1 if protocols are well-documented<br>+ 1 if public protocol is used
	2	**Multiple segmentations** - possible actions are: segmentation by different physicians/algorithms/software, perturbing segmentations by (random) noise, segmentation at different breathing cycles. Analyse feature robustness to segmentation variabilities	+ 1 if segmented multiple times (different physicians, algorithms, or perturbation of regions of interest)
	3	**Phantom study on all scanners** - detect inter-scanner differences and vendor-dependent features. Analyse feature robustness to these sources of variability	+ 1 if texture phantoms were used for feature robustness assessment
	4	**Imaging at multiple time points** - collect images of individuals at additional time points. Analyse feature robustness to temporal variabilities (for example, organ movement, organ expansion/ shrinkage)	+ 1 multiple time points for feature robustness assessment
**Domain 2:** Feature selection and validation (-8 to 8)	5	**Feature reduction or adjustment for multiple testing** - decreases the risk of overfitting. Overfitting is inevitable if the number of features exceeds the number of samples. Consider feature robustness when selecting features	- 3 if neither measure is implemented<br>+ 3 if either measure is implemented
	12	**Validation** - the validation is performed without retraining and without adaptation of the cut-off value, provides crucial information with regard to credible clinical performance	− 5 if validation is missing<br>+ 2 if validation is based on a dataset from the same institute/<br>+ 3 if validation is based on a dataset from another institute/<br>+ 4 if validation is based on two datasets from two distinct institutes/<br>+4 if the study validates a previously published signature/<br>+5 if validation is based on three or more datasets from distinct institutes<br>*Datasets should be of comparable size and should have at least 10 events per model feature
**Domain 3:** Biologic/clinical validation and utility (0 to 6)	6	**Multivariable analysis with non-radiomics features** (for example, EGFR mutation) - is expected to provide a more holistic model. Permits correlating/inferencing between radiomics and non-radiomics features	+ 1 if multivariable analysis with non-radiomics features
	7	**Detect and discuss biological correlates** - demonstration of phenotypic differences (possibly associated with underlying gene–protein expression	+ 1 if present

		patterns) deepens understanding of radiomics and biology	
	13	**Comparison to gold standard** - assess the extent to which the model agrees with/is superior to the current 'gold standard' method (for example, TNM-staging for survival prediction). This comparison shows the added value of radiomics	+ 2 for comparison to gold standard
	14	**Potential clinical utility** - report on the current and potential application of the model in a clinical setting (for example, decision curve analysis)	+ 2 for reporting potential clinical utility
**Domain 4:** Model performance index (0 to 5)	8	**Cut-off analyses** - determine risk groups by either the median, a previously published cut-off or report a continuous risk variable. Reduces the risk of reporting overly optimistic results	+ 1 if cutoff either pre-defined or at median or continuous risk variable reported
	9	**Discrimination statistics** - report discrimination statistics (for example, C-statistic, ROC curve, AUC) and their statistical significance (for example, p-values, confidence intervals). One can also apply resampling method (for example, bootstrapping, cross-validation)	+ 1 if a discrimination statistic and its statistical significance are reported<br>+ 1 if a resampling method technique is also applied
	10	**Calibration statistics** - report calibration statistics (for example, Calibration-in-the-large/slope, calibration plots) and their statistical significance (for example, *P*-values, confidence intervals). One can also apply resampling method (for example, bootstrapping, cross-validation)	+ 1 if a calibration statistic and its statistical significance are reported<br>+ 1 if a resampling method technique is also applied
**Domain 5:** High level of evidence (0 to 8)	11	**Prospective study registered in a trial database** - provides the highest level of evidence supporting the clinical validity and usefulness of the radiomics biomarker	+ 7 for prospective validation of a radiomics signature in an appropriate trial
	15	**Cost-effectiveness analysis** - report on the cost-effectiveness of the clinical application (for example, QALYs generated)	+ 1 for cost-effectiveness analysis
**Domain 6:** Open science and data (0 to 4)	16	**Open science and data** - make code and data publicly available. Open science facilitates knowledge transfer and reproducibility of the study	+ 1 if scans are open source<br>+ 1 if region of interest segmentations are open source<br>+ 1 if code is open source<br>+ 1 if radiomics features are calculated on a set of representative ROIs and the calculated features and representative ROIs are open source
	Total points (36 = 100%)		

Note: RQS = Radiomics Quality Score.

Extracted from Lambin P, Leijenaar RTH, Deist TM, et al. Radiomics: the bridge between medical imaging and personalized medicine. Nat Rev Clin Oncol. 2017;14(12):749-762.

Supplementary Table S3 Reporting Completeness according to TRIPOD Statement

Section	TRIPOD#	Item	Explanation
**Title and Abstract**	1	**Title** - identify developing/validating a model, target population, and the outcome	#1: considered as complete if all elements of the type of study (development, validation, incremental value or combination), the target population, and outcome are included.
	2	**Abstract** - provide a summary of objectives, study design, setting, participants, sample size, predictors, outcome, statistical analysis, results, and conclusions	#2 and #3b: considered as complete if 'development' and/or 'validation' is explicitly written. Synonyms instead of development such as 'establish', 'build', 'investigate', and 'evaluate' were not considered as complete.
**Introduction**	3a	**Background** - Explain the medical context and rationale for developing/validating the model	#3a: considered as complete if at least a simple sentence was provided to introduce the medical context and rationale for developing/validating the model.
	3b	**Objective** - Specify the objectives, including whether the study describes the development/validation of the model or both.	#2 and #3b: considered as complete if 'development' and/or 'validation' is explicitly written. Synonyms instead of development such as 'establish', 'build', 'investigate', and 'evaluate' were not considered as complete
**Methods**	4a	**Source of data** - describe the study design or source of data (randomized trial, cohort, or registry data)	#4a: whether the study was conducted in a randomized controlled trial, cohort, or registry with a consecutive, random, or convenience series. A study was considered as complete when the terms 'retrospective' or 'prospective' were mentioned.
	4b	**Source of data** - specify the key dates	#4b: provide the name of open-source data, or declaim that the study was performed based on institutional dataset with a specific inclusion period.
	5a	**Participants** - specify key elements of the study setting including number and location of centers	#5a: number and location of centers should be declared in multicenter studies; monocenter study should state the location of that the study performed.
	5b	**Participants** - describe eligibility criteria for participants (inclusion and exclusion criteria)	#5b: considered as complete if a structured criterion of inclusion and exclusion were provided; only disease name was not considered as complete.
	5c	**Participants** - give details of treatment received, *if relevant*	#5c: treatments are relevant in prognostic studies as they modify outcomes and relevant information should be reported.
	6a	**Outcome** - clearly define the outcome, including how and when assessed	#6a: the method of assessment, e.g., histology and experience of pathologists; follow-up, frequency and modality; or expert's opinion and experience of experts.
	6b	**Outcome** - report any actions to blind assessment of the outcome	#6b: describe weather the outcome is ideally assessed while blinded to information about the predictors.
	7a	**Predictors** - clearly define all predictors, including how and when assessed	#7a: the radiomics studies involve quantitative feature extraction through an automated process; thus, the element 'when' was ignored.

	7b	**Predictors** - report any actions to blind assessment of predictors for the outcome and other predictors	#7b: if radiomics studies were based on regions-of-interest and the blindness of readers to the reference standard was considered, they were recorded as complete. If ‘blind’ or ‘unaware of’ the reference standard was not explicitly written, it was considered as incomplete. Automatic segmentation was considered as complete.
	8	**Sample size** - explain how the study size was arrived at	#8: considered as complete if the database, software or method, and results were described.
	9	**Missing data** - describe how missing data were handled with details of any imputation method	#9: considered as complete if the imputation method was described when there is missing data, or how to excluded the insufficient data when imputation was not performed
	10a	**Statistical analysis methods** - describe how predictors were handled	#10a: considered as complete if the statistical analysis method (e.g., t test, chi-square test) were included, and suitable for the variable type.
	10b	**Statistical analysis methods** - specify type of model, all model-building procedures (any predictor selection), and method for internal validation	#10b: considered as complete if all three elements, model type (e.g., logistic regression, Cox proportional hazards model), feature selection procedure to control overfitting, and methods of internal validation (cross-validation, bootstrap sample), were included. A regularization or penalization method such as the least absolute shrinkage and selection operator (LASSO) was considered as both a feature selection procedure and internal validation, as it contains 10-fold cross-validation as a default setting.
	10d	**Statistical analysis methods** - specify all measures used to assess model performance and if relevant, to compare multiple models (discrimination or calibration)	#10d: the article was considered as complete if both the discrimination or calibration index were written
	11	**Risk groups** - provide details on how risk groups were created, if done	#11: considered as complete if the cutoffs were provided, e.g., disease stage, predictive absolute incidence, or risk rate.
**Results**	13a	**Participants** - describe the flow of participants, including the number of participants with and without the outcome. A diagram may be helpful.	#13a: considered as complete if a diagram or text description with the numbers of screened patients, excluded patients and included patients was provided.
	13b	**Participants** - describe the characteristics of the participants, including the number of participants with missing data for predictors and outcome	#13b: considered as complete if a table or text description was provided.
	14a	**Model development** - specify the number of participants and outcome events in each analysis	#14a: considered as complete if a table or text description was provided.
	14b	**Model development** - report the unadjusted	#14b: considered as complete if the metrics and their confidence interval

		association between each candidate predictor and outcome, *if done*	were provided.
	15a	**Model specification** - present the full prediction model to allow predictions for individuals (regression coefficients, intercept)	#10c, #15a, and #15b: these items determine if an article describes how the obtained model predicted the outcome probabilities for an individual. If the articles described this in the methods (item 10c) and contained a full prediction model including all regression coefficients and the intercept or baseline hazard for a particular time point, they were considered as complete for item 15a. If the study contained explicit formula or a nomogram, the study was considered as complete for item 15b.
	15b	**Model specification** - explain how to the use the prediction model (nomogram, calculator, etc)	#10c, #15a, and #15b: these items determine if an article describes how the obtained model predicted the outcome probabilities for an individual. If the articles described this in the methods (item 10c) and contained a full prediction model including all regression coefficients and the intercept or baseline hazard for a particular time point, they were considered as complete for item 15a. If the study contained explicit formula or a nomogram, the study was considered as complete for item 15b.
	16	**Model performance** - report performance measures (with confidence intervals) for the prediction model	#16: considered as complete if the metrics (at least the discrimination outcome) and their confidence interval were provided.
**Discussion**	18	**Limitations** - Discuss any limitations of the study	#18: considered as complete if there was a limitation paragraph, usually the paragraph before the conclusion.
	19b	**Interpretation** - Give an overall interpretation of the results	#19b: considered as complete if there was an interpretation of results paragraph, usually the paragraph of discussion.
	20	**Implications** - Discuss the potential clinical use of the model and implications for future research	#20: considered as complete if there was text description or decision curve analysis. This is different from 'clinical validity' in RQS criterion 14, that the decision curve analysis was necessary.
**Validation (types 2a, 2b, 3, and 4)**	10c	**Statistical analysis methods** - describe how the predictions were calculated	#10c, #15a, and #15b: these items determine if an article describes how the obtained model predicted the outcome probabilities for an individual. If the articles described this in the methods (item 10c) and contained a full prediction model including all regression coefficients and the intercept or baseline hazard for a particular time point, they were considered as complete for item 15a. If the study contained explicit formula or a nomogram, the study was considered as complete for item 15b.
	10e	**Statistical analysis methods** - describe any model updating (recalibration), *if done*	#10e and #17: If an article describes methods to adjust (recalibrate) or update a previously developed prediction model, the article is scored. This is different from 'comparison with gold standard' in RQS criterion 13, in that it requires recalibration of regression coefficients and hazard ratios in the pre-existing model, and was scored if it was completely reported.

	12	**Development vs. validation** - Identify any differences from the development data in setting, eligibility criteria, outcome, and predictors	#12 and #13c: considered as complete if a table comparing developing and testing dataset or text description was provided.
	13c	**Participants (for validation)** - show a comparison with the development data of the distribution of important variables	#12 and #13c: considered as complete if a table comparing developing and testing dataset or text description was provided.
	17	**Model updating** - report the results from any model updating, *if done*	#10e and #17: If an article describes methods to adjust (recalibrate) or update a previously developed prediction model, the article is scored. This is different from 'comparison with gold standard' in RQS item 13, in that it requires recalibration of regression coefficients and hazard ratios in the pre-existing model, and was scored if it was completely reported.
	19a	**Interpretation (for validation)** - discuss the results with reference to performance in the development data and any other validation data	#19a: considered as complete if there was a paragraph that discuss the influence of difference between development and validation data on the model performance. The performance of the model in the validation study should be discussed and placed in context to the model performance in the original development study and with any other existing validation studies of that model. One should highlight the main results, as well as any biases that may have affected the comparison.<br>When the validation study shows a different (usu- ally poorer) performance, reasons should be discussed to enhance interpretation.
**Other Information**	21 (Excluded)	**Supplementary information** - provide information about the availability of supplementary resources, such as study protocol, Web calculator, and data sets	#21: considered as complete if the study provided supplementary materials and/or links for online resources, or declared that all data were provided in the manuscript.
	22 (Excluded)	**Funding** - give the source of funding and the role of the funders for the present study	#22: considered as complete if the source of funding and the role of the funders were both declared.

Note: TRIPOD = Transparent Reporting of a multivariable prediction model for Individual Prognosis Or Diagnosis.
Extracted from Collins GS, Reitsma JB, Altman DG, Moons KG. Transparent reporting of a multivariable prediction model for individual prognosis or diagnosis (TRIPOD): the TRIPOD statement. Ann Intern Med. 2015;162(1):55-63.

Supplementary Table S4 Pre-processing Steps according to IBSI Guideline

IBSI#	Pre-Processing Performed	Explanation
46	**Intensity normalization** - describe the method and settings used to normalize intensity distributions within a patient or patient cohort.	Any kind of normalization method was accepted, such as white stripe normalization, z-score normalization, or normalization using the $\mu \pm 3\sigma$ method.
48	**Segmentation method** - describe how regions of interest were segmented; describe the number of experts, their expertise and consensus strategies for manual delineation; describe methods and settings used for semi-automatic and fully automatic segmentation; describe which image was used to define segmentation in case of multi-modality imaging.	Any kind of segmentation method was accepted, such as manual segmentation, semi-automatic segmentation, or fully automatic segmentation, with or without providing number of experts, their expertise and consensus strategies for manual delineation, or settings used for semi-automatic or fully automatic segmentation.
50	**Image interpolation (iso-voxel resampling)** - describe which interpolation algorithm was used to interpolate the image; describe how the position of the interpolation grid was defined; describe how the dimensions of the interpolation grid were defined; describe how extrapolation beyond the original image was handled.	Mentioning the exact term "interpolation" or "resampling" was presumed to perform iso-voxel resampling with or without providing interpolation algorithm, the position of the interpolation grid, or how extrapolation beyond the original image was handled.
56	**Grey-level discretization** - describe the method used to discretize image intensities.	Mentioning the exact term "discretization" was presumed to perform gray-level discretization with or without providing the number of bins or the size of the bins.
57	**Image filter** - describe whether and which methods and settings were used to filter images.	Any kind of filtering method was accepted, such as Laplacian-of-Gaussian, wavelet, or a declaration of non-filtering.
59	**IBSI compliance** - state if the software used to extract the set of image biomarkers is able to reproduce the IBSI feature reference values.	A software is compliant if and only if it is able to reproduce image biomarker reference values for the digital phantom and for one or more image processing configurations using the radiomics CT phantom. We documented the name of software, and then found out whether they were IBSI compliant or not.
60	**Robustness** - describe how robustness of the image biomarkers was assessed.	Robustness is one of the key concerns for generalizability and application of radiomics models. We documented the method of robustness assessment, e.g. test-retest analysis, before the model building.

Note: IBSI = Image Biomarker Standardization Initiative.
Extracted from Zwanenburg A, Vallières M, Abdalah MA, et al. The Image Biomarker Standardization Initiative: Standardized Quantitative Radiomics for High-Throughput Image-based Phenotyping. Radiology. 2020;295(2):328-338.

Supplementary Table S5 Risk of Bias and Concern on Application Assessment according to QUADAS-2 Tool

Domain and Description	Modified signaling question	Risk of bias	Applicability concern
**Patient selection** - describe methods of patient selection: Describe included patients (prior testing, presentation, intended use of index test and setting)	Signaling question 1: was a consecutive or random sample of patients enrolled?	Could the selection of patients have introduced bias?	Are there concerns that the included patients do not match the review question?
	Signaling question 2: was a case–control design avoided?		
	Signaling question 3: did the study avoid inappropriate exclusions?		
**Index test** - describe the index test and how it was conducted and interpreted	Signaling question 1: were the imaging acquisition protocol, image processing approach described in detail?	Could the conduct or interpretation of the index test have introduced bias?	Are there concerns that the index test, its conduct, or interpretation differ from the review question?
	Signaling question 2: were the segmentation method(s), and feature extraction software described in detail?		
	Signaling question 3: was the validation independent (i. e. external)?		
**Reference standard** - describe the reference standard and how it was conducted and interpreted	Signaling question 1: is the reference standard likely to correctly classify the target condition?	Could the reference standard, its conduct, or its interpretation have introduced bias?	Are there concerns that the target condition as defined by the reference standard does not match the review question?
**Flow and timing** - describe any patients who did not receive the index test(s) and/or reference standard or who were excluded from the 2x2 table (refer to flow diagram): Describe the time interval and any interventions between index test(s) and reference standard	Signaling question 1: was there an appropriate interval between imaging and reference standard?	Could the patient flow have introduced bias?	-

Note: QUADAS-2 = modified Quality Assessment of Diagnostic Accuracy Studies

Extracted from Whiting PF, Rutjes AW, Westwood Me, et al; QUADAS-2 Group. QUADAS-2: a revised tool for the quality assessment of diagnostic accuracy studies. Ann Intern Med. 2011;155(8):529-536.

Supplementary Table S6 Types of Prediction Model Studies Covered by The TRIPOD Statement

**Model type**	**Description**
Type 1a	Development of a prediction model where predictive performance is then directly evaluated using exactly the same data (apparent performance).
Type 1b	Development of a prediction model using the entire data set, but then using resampling (e.g., bootstrapping or cross-validation) techniques to evaluate the performance and optimism of the developed model. Resampling techniques, generally referred to as "internal validation", are recommended as a prerequisite for prediction model development, particularly if data are limited.
Type 2a	The data are randomly split into 2 groups: one to develop the prediction model and one to evaluate its predictive performance. This design is generally not recommended or better than type 1b, particularly in case of limited data, because it leads to lack of power during model development and validation.
Type 2b	The data are nonrandomly split (e.g., by location or time) into 2 groups: one to develop the prediction model and one to evaluate its predictive performance. Type 2b is a stronger design for evaluating model performance than type 2a because it allows for nonrandom variation between the 2 data sets.
Type 3	Development of a prediction model using 1 data set and an evaluation of its performance on separate data (e.g., from a different study).
Type 4	The evaluation of the predictive performance of an existing (published) prediction model on separate data.

Note: TRIPOD = Transparent Reporting of a multivariable prediction model for Individual Prognosis Or Diagnosis.

Extracted from Collins GS, Reitsma JB, Altman DG, Moons KG. Transparent reporting of a multivariable prediction model for individual prognosis or diagnosis (TRIPOD): the TRIPOD statement. Ann Intern Med. 2015;162(1):55-63.

Supplementary Table S7 Trials Classifications for Image Mining Tools Development Process

Trial phase	Sample size	Type of study	Validation approach	Development stage
Discovery science	Algorithm development, phantom, simulated data	Experimental	Without validation	Preclinical
Phase 0	< 100 patients	Retrospective	Internal validation	Pre-marketing
Phase I	< 100 patients	Retrospective	Validation	Pre-marketing
Phase II	> 100 patients	Retrospective	Validation	Pre-marketing
Phase III	> 100 patients	Prospective	Validation	Pre-marketing
Phase IV	n/a	Real-world	Monitoring	Post-marketing

Note: The validation process may be internal (e.g., cross-validation and bootstrapping) or external (using data not used for training). The external validation may be performed using three different strategies: (i) temporal (i.e., data obtained in newly recruited patients), (ii) geographic (i.e., data collected in a different institution), and (iii) split-sample (i.e., data split from the entire dataset and kept untouched for the test).

Extracted form Sollini M, Antunovic L, Chiti A, Kirienko M. Towards clinical application of image mining: a systematic review on artificial intelligence and radiomics. Eur J Nucl Med Mol Imaging. 2019;46(13):2656-2672.

Supplementary Table S8 Category of Five Levels of Supporting Evidence of Meta-analyzes

Levels of Supporting Evidence	Description
Convincing	$p < 10^{-6}$, > 1000 events, the largest study reaches statistical significance ($p < 0.05$), $I2 < 50\%$, the null value excluded by the 95% PI, no small-study effects ($p > 0.1$ for Egger's test) and excess significance ($p > 0.1$), and survived the 10% credibility ceiling ($p < 0.05$)
Highly Suggestive	$p < 10^{-6}$, > 1000 events, the largest study reaches statistical significance ($p < 0.05$)
Suggestive	$p < 10^{-3}$, > 1000 events
Weak	$p < 0.05$
Not Suggestive	$p > 0.05$

Note: Extracted from Dang Y, Hou Y. The prognostic value of late gadolinium enhancement in heart diseases: an umbrella review of meta-analyses of observational studies. Eur Radiol. 2021;31(7):4528-4537.

Supplementary Table S9 Study Characteristics of Included Studies

Study	Country	Journal	Impact Factor	Journal Type	First Authorship	Study Design	Imaging Modality	Biomarker	Clinical question
Chen2019	China	Eur Radiol	5.315	Imaging	Radiologist	Retrospective	CT	Prognostic	AP (recurrence)
Cheng2018	China	Sci Rep	4.380	Non-imaging	Radiologist	Retrospective	PET	Diagnostic	AIP vs. PC
Cui2021	China	Chin J Dig Surg	n/a	Non-imaging	Radiologist	Retrospective	CT	Prognostic	AP (recurrence)
Das2008	USA	Gastrointest Endosc	9.427	Non-imaging	Non-radiologist	Retrospective	EUS	Diagnostic	CP vs. PC vs. normal tissue
Deng2021	China	Front Oncol	6.244	Non-imaging	Radiologist	Retrospective	MRI	Diagnostic	MFP vs. PC
E2020	China	J Comput Assist Tomogr	1.826	Imaging	Radiologist	Retrospective	CT	Diagnostic	AIP vs. PC
Frøkjær2020	Denmark	Abdom Radiol (NY)	3.039	Imaging	Radiologist	Prospective	MRI	Diagnostic	CP vs. normal tissue
Hu2021	China	Chin J Magn Reson Imaging	n/a	Imaging	Radiologist	Retrospective	MRI	Prognostic	AP (recurrence)
Iranmahboob2017	USA	Clin Imaging	1.605	Imaging	Radiologist	Retrospective	MRI	Prognostic	AP (progression)
Li2021	China	J Chin Clin Med Imaging	n/a	Imaging	Radiologist	Retrospective	CT	Diagnostic	AIP vs. PC
Li2022	China	Acad Radiol	3.173	Imaging	Radiologist	Retrospective	CT	Diagnostic	AIP vs. PC
Lin2019	China	J Pract Radiol	n/a	Imaging	Radiologist	Retrospective	CT	Diagnostic	AIP vs. PC
Lin2020	China	J Magn Reson Imaging	4.813	Imaging	Radiologist	Retrospective	MRI	Prognostic	AP (severity)
Liu2021	China	Eur Radiol	5.315	Imaging	Non-radiologist	Retrospective	PET	Diagnostic	AIP vs. PC
Liu2022	China	Transl Oncol	4.243	Non-imaging	Radiologist	Retrospective	MRI	Diagnostic	MFP vs. PC
Ma2022	China	Int J Gen Med	2.466	Non-imaging	Radiologist	Retrospective	CT	Diagnostic	CP vs. PC
Mashayekhi2020	USA	Eur J Radiol	3.528	Imaging	Radiologist	Retrospective	CT	Diagnostic	CP vs. recurrent AP vs. functional abdominal pain
Park2020	USA	Diagn Interv Imaging	4.026	Imaging	Radiologist	Retrospective	CT	Diagnostic	AIP vs. PC

Peng2020	China	Chin J Magn Reson Imagin	n/a	Imaging	Radiologist	Retrospective	MRI	Diagnostic	MFP vs. PC; MFP vs. SPT + pNET
Ren2019	China	Front Oncol	6.244	Non-imaging	Radiologist	Retrospective	CT	Diagnostic	MFP vs. PC
Ren2020	China	Abdom Radiol (NY)	3.039	Imaging	Radiologist	Retrospective	CT	Diagnostic	MFP vs. PC
Ren2021	Japan	Abdom Radiol (NY)	3.039	Imaging	Radiologist	Retrospective	MRI	Diagnostic	MFP vs. PC
Zhang2010	China	Gastrointest Endosc	9.427	Imaging	Radiologist	Retrospective	EUS	Diagnostic	CP vs. normal tissue
Zhang2019A	China	Med Phys	4.071	Non-imaging	Non-radiologist	Retrospective	PET	Diagnostic	AIP vs. PC
Zhang2019B	China	J Biomedical Eng	n/a	Non-imaging	Non-radiologist	Retrospective	PET	Diagnostic	AIP vs. PC
Zhang2019C	China	Natl Med J China	n/a	Non-imaging	Radiologist	Retrospective	CT	Diagnostic	MFP vs. PC
Zhou2021	China	Pancreas	3.327	Non-imaging	Radiologist	Retrospective	MRI	Prognostic	AP (extrapancreatic necrosis)
Zhu2013	China	PLoS One	3.240	Non-imaging	Non-radiologist	Retrospective	EUS	Diagnostic	CP vs. PC
Zhu2015	China	Gastrointest Endosc	9.427	Non-imaging	Non-radiologist	Retrospective	EUS	Diagnostic	AIP vs. CP
Ziegelmayer2020	Germany	J Clin Med	4.242	Non-imaging	Radiologist	Retrospective	CT	Diagnostic	AIP vs. PC

Note: PC = pancreatic cancer, CP = chronic pancreatitis, AIP = autoimmune pancreatitis, MFP = mass-forming pancreatitis, AP = acute pancreatitis, SPT = solid-pseudopapillary tumor of pancreas, pNET = pancreatic neuroendocrine tumor.

Supplementary Table S10 PICOT of Included Studies

Study	Sample Size	Institution	Inclusion Period	Patient Condition	Gender (F/M)	Age	Imaging	Comparing Test	Reference Standard	Timing
Chen2019	389	3 centers	Jan 2010 to Jun 2014	208 AP patients: admitted with a first attack; 181 RAP patients: AP patients admitted with a first attack and were followed up with a documented readmission for AP.	151/238	Mean ± SD, 49.20 ± 15.44	CT	Clinical model	Clinical criteria, Follow up from January 2010 to May 2018; follow-up period 62.8 ± 6.7 mo.	Within 1 week of symptom onset
Cheng2018	114	1 center	Sep2008 to Jul 2016	53 patients with suspected AIP according to the 2008 Asian Diagnostic Criteria; 61 patients later pathologically proven have PC during the same study period. 3 suspected AIP patients were confirmed as PC; therefore, 51 AIP patients fit 2011 International Consensus Diagnostic Criteria for AIP and 64 pathologically proven PC	6/47 for AIP; 28/33 for PC	63.0 ± 14.0; 65.0 ± 15.0	PET	None	Clinical criteria or histology; Histology	All conventional imaging studies and procedures were performed within 2 weeks of PET/CT.

				patients.						
Cui2021	56	1 center	Jan 2016 to Jan 2018	56 pediatric AP patients: admitted with a first attack, 20 patients were followed up with a documented readmission for AP.	43/13	Median 5.5, range 3.5 to 13.0	CT	None	Follow-up ≥ 24 mo.	Within 24h of admission.
Das2008	56	1 center	NR	22 patients with a normal pancreas underwent EUS for indications unrelated to the pancreas; 12 CP patients diagnosed on the basis of their clinical presentation and at least two different imaging modalities that showed the characteristic features of CP, in addition to EUS findings of CP; 22 PC patients established by EUS-guided FNA.	NR	NR	EUS	None	Clinical criteria or histology; Histology	NR
Deng2021	119	2 centers	Mar 2016 to Jun 201	13/10 patients had a definitive diagnosis of MCP defined as	17/47 for center 1; 26/29 for center 2	Median 60/63 for MCP/PC in center 1;	MRI	Clinical model	Histology; histology	NR

				chronic inflammation with focal mass formation confirmed; 51/45 patients had a definitive diagnosis of PDAC confirmed by histopathology.		57/62 for MCP/PC in center 2				
E2020	96	2 centers	Jan 2012 to May 2019	45 patients with focal-type AIP, including 14 patients confirmed with a histologic diagnosis and 31 patients who met the ICDC; 51 PC patients was matched to the patients with AIP	19/26 for AIP; 13/38 for PC	Median 61 for AIP, 68 for PC	CT	Radiologists' assessment	Clinical criteria or histology; Histology	NR
Frøkjær2020	99	1 center	NR	77 patients diagnosed based on Lüneburg criteria, where CP was defined as a score ≥ 4 points; 22 healthy control without history of pancreas-related diseases or gastrointestinal diseases	21/51 for CP; 10/12 for HC	59.8 ± 10.4 for CP; 54.2 ± 6.8 for HC	MR	None	Clinical criteria; Histology	NR
Hu2021	147	1 center	Jan 2014 to Dec	102 AP patients: admitted with a	49/53 for AP;	47.33±14.85 for AP;	MR	None	Clinical criteria, follow up	NR

			2015	first attack; 45 RAP patients: AP patients admitted with a first attack and were followed up with a documented readmission for AP.	14/31 for RAP	46.49±14.05 for RAP				
Iranmahboob2017	41	1 center	2006 to 2014	41 AP patients	16/25	57 ± 17	MR	None	Median follow-up cross-sectional imaging was 28 days (inter-quartile range, 68 days); Radiologists' assessment	NR
Li2021	110	1 center	Jan 2013 to Jun 2020	50 AIP by Chinese clinical criteria; 60 PC confirmed by surgery or biopsy histology	NR	63.7 ± 11.9	CT	Radiologists' assessment	Clinical criteria; Histology	Within 30d of surgery
Li2022	97	1 center	Jan 2011 to Dec 2018	42 AIP according to the International Consensus Diagnostic Criteria; 55 PC pathologically confirmed	9/33 for AIP; 14/41 for PC	60.79 ± 10.37 for AIP; 60.07 ± 9.81 for PC	CT	None	Clinical criteria; Histology	NR
Lin2019	56	1 center	Jun 2014 to Feb 2018	25 AIP according to the International Consensus Diagnostic Criteria; 31 PC	14/42	53 ± 12	CT	None	Clinical criteria; Histology	NR

				pathologically confirmed						
Lin2020	259	1 center	Jan 2016 to Oct 2018	142 patients with mild acute pancreatitis (MAP), 91 patients with moderately severe acute pancreatitis (MSAP), and 26 patients with severe acute pancreatitis (SAP)	76/104 for training; 33/46 for validation	49.39 ± 14.12; 48.43 ± 15.65	MR	APACHE II score, BISAP score, and MRSI	Clinical criteria	not more than 3 days from AP onset to MRI examination
Liu2021	112	1 center	Feb 2011 to Jun 2019	48 AIP according to 14th International Pancreatology Conference, in which 25 diagnosed with histology; 64 PC confirmed by histology or cytology	5/43 for AIP; 29/35 for PC	Median 66, (range, 37 to 90) for AIP; 66.5 (32 to 88) for PC	PET/CT	Radiologists' assessment	Clinical criteria or histology; Histology	NR
Liu2022	102	1 center	Jan 2017 to Mar 2021	65 MFP confirmed by histology or cytology; 81 PC confirmed by histology	17/21 for PC training; 18/16 for MFP training; 5/11 for PC validation; 6/8 for MFP validation	61.6 ± 14.4 for PC training; 62.16 ± 14.1 for MFP training; 63.3 ± 13.5 for PC validation; 60.5 ± 11.5 for MFP	MRI	Clinical	Histology; histology	NR

						validation				
Ma2022	175	3 centers	Jan 2018 to Oct 2020	151 patients with PC and 24 patients comprehensively diagnosed with CP (18 patients with AIP and 6 patients of MFP)	5/19 for CP; 66/85 for PC	Median 65 (IQR 60, 70) for CP; 64 (60, 70) for PC	CT	Clinical	Comprehensively diagnosed; comprehensively diagnosed	NR
Mashayekhi2020	56	1 center	2010 to 2018	20 RAP patients according to criteria of the revised Atlanta classification; 19 patients with functional abdominal pain defined using the ROME IV criteria; 17 CP patients defined according to definite M-ANNHEIM criteria.	9/11 for RAP; 12/7 for functional abdominal pain; 8/7 for CP	44.9 ± 13.7 for RAP; 46.2 ± 11.3 for functional abdominal pain; 56.1 ± 13.4 for CP	CT	None	Clinical criteria; Clinical criteria; Clinical criteria or histology	NR
Park2020	182	1 center	2014 to 2018	89 AIP patients diagnosed according to clinical criteria, biopsy or histology; 93 PC patients confirmed by histology	24/65 for AIP; 25/68 for PC	59.7 ± 13.9 for AIP; 60.1 ± 12.3 for PC	CT	None	Clinical criteria or histology; Histology	NR
Peng2020	177	1 center	Jun 2016 to Jun 2018	69 PC patients; 12 SPT patients; 15 pNET patients; 21 MFP	51/66	57.8±12.1	MRI	None	Histology; Histology; Histology; Histology	NR

				patients. All confirmed by histology.						
Ren2019	109	1 center	Jan 2012 to Dec 2017 for MFP; Jan 2015 to Dec 2017 for PC	30 MFP patients proved by surgery or biopsy; 79 PC patients proved by surgery or biopsy	5/25 for MFP; 23/56 for PC	61.47 ± 12.43 for MFP; 65.18 ± 8.60 for PC	CT	Radiologists' assessment	Histology; Histology	< 30 days prior to resection or biopsy
Ren2020	109	1 center	Jan 2012 to Dec 2017	30 MFP patients proved by surgery or biopsy; 79 PC patients proved by surgery or biopsy	5/25 for MFP; 23/56 for PC	61.47 ± 12.43 for MFP; 65.18 ± 8.60 for PC	CT	None	Histology; Histology	NR
Ren2021	167	1 center	Apr 2008 to Aug 2020	23 AIP patients diagnosed by Japan Pancreas Society (JPS) 2018 diagnostic criteria, 22 patients based on the histological findings of endoscopic ultrasound-guided fine-needle aspiration (EUS-FNA), 1 diagnosed by clinical criteria; 144 PC patients diagnosed by surgery or biopsy	4/19 for AIP;62/82 for PC	Median 64 (range, 47 to 81) for AIP; 68 (41 to 92) for PC	MRI	None	Clinical criteria or histology; Histology	The median time interval between MRI examination and histological diagnosis of PDAC was 10 days (range 0–75 days).

Zhang2010	216	1 center	Mar 2005 to Dec 2007	153 PC; 63 noncancer (20 normal tissue, 43 CP). All confirmed by EUS-guided FNA and pathologic analysis.	NR	NR	EUS	None	Histology; Histology	NR
Zhang2019A	111	1 center	Feb 2011 to Nov 2017	45 AIP patients were confirmed based on the 14th International Association of Pancreatology diagnostic criteria; 66 PDAC patients were confirmed based on histological findings or liquid based cytology	4/41 for AIP; 30/36 for PC	Median 60, (range, 34 to 87) for AIP; 61, (24 to 82) for PC	PET/CT	Radiologists' assessment	Clinical criteria or histology; Histology	NR
Zhang2019B	111	1 center	NR	45 AIP; 66 PDAC. All confirmed by histology or biopsy	NR	NR	PET/CT	None	Histology; Histology	NR
Zhang2019C	68	2 centers	Jan 2014 to Dec 2017	21 MFP confirmed by biopsy and follow-up; 47 PC confirmed by surgery	4/17 for MFP; 12/35 for PC	61 ± 13 for MFP; 65 ± 9 for PC	CT	Radiologists' assessment	Histology; Histology	NR
Zhou2021	135	1 center	Sep 2014 to Sep 2019	135 first-episode AP. A patient was included in the EXPN group	63/72	49.8 ± 14.5	MRI	Radiologists' assessment	Follow-up	within 7 days of onset with extra-pancreatic

				when conforming to follow-up MRI or CT or histopathological findings						collections visible on MRI and were followed up with either MRI or CT after 7 days or with histopathological findings
Zhu2013	388	1 center	May 2022 to Aug 2011	126 CP patients diagnosed by standard CP Sahai diagnostic criteria and follow-up; 262 PC patients confirmed by histology	NR	NR	EUS	None	Clinical criteria; Histology	NR
Zhu2015	181	1 center	May 2005 to Jan 2013 for AIP; Aug 2008 to Jan 2013 for CP	81 AIP diagnosed the Mayo Clinic's HISORt criteria; 100 CP diagnosed according to the EUS criteria defined by Sahai	NR	NR	EUS	None	Clinical criteria; Clinical criteria	NR
Ziegelmayer2020	86	1 center		44 patients with AIP either diagnosed histopathologically or based on a combination of serology, imaging data, and therapy response to corticosteroids in	15/29 for AIP; 23/19 for PC	57 ± 17.3 for AIP; 67 ± 10.6 for PC	CT	Noen	Clinical criteria or histology; Histology	NR

				accordance with the international consensus diagnostic criteria (ICDC) ;42 patients with PC diagnosed histopathologically						

Note: PICOT = population, intervention, control, outcome and timing. PC = pancreatic cancer, CP = chronic pancreatitis, AIP = autoimmune pancreatitis, MFP = mass-forming pancreatitis, AP = acute pancreatitis, SPT = solid-pseudopapillary tumor of pancreas, pNET = pancreatic neuroendocrine tumor.

Supplementary Table S11 Radiomics Methodological Consideration of Included Studies

Study	Imaging	ROI segmentation	Radiomics feature extraction	Feature reduction and selection	Classifier	Outcome	Validation	Model type	Phase
Chen2019	CT (arterial and portal)	2 readers; blinded	IBEX on MATLAB	(1) samples t test or the Mann-Whitney U test; (2) least absolute shrinkage and selection operator (LASSO); (3) Spearman correlation coefficients.	(1) logistic regression (LR); (2) support vector machine (SVM)	AP (recurrence)	randomly split-sample	2a	II
Cheng2018	PET/CT (FDG)	2 readers; blinded	NR	(1) Student t test; (2) univariate and multivariate analyses; (3) backward stepwise logistic regression analysis.	logistic regression (LR)	AIP vs. PC	the same data	1a	0
Cui2021	CT (non-enhanced, arterial and portal)	2 readers; blinded	Omni-Kinetics	(1) samples t test or the Mann-Whitney U test; (2) multivariate analyses.	logistic regression (LR)	AP (recurrence)	the same data	1a	0
Das2008	EUS	NR; not blinded	Image J	principal component analysis	artificial neural network (ANN)	CP vs. PC vs. normal tissue	randomly split-sample	2a	0
Deng2021	MRI (T1WI, T2WI, DCE)	2 readers; blinded	IBEX on MATLAB	(1) samples t test or the Mann-Whitney U test; (2) univariate analysis and least absolute shrinkage and se-lection operator (LASSO)	support vector machine (SVM)	MFP vs. PC	geographical	2b	II
E2020	CT (non-enhanced, arterial and portal)	1 reader; blinded; 1 reader for robustness	in-house software on MATLAB	coarse-to-fine 2-stage strategy	random forest (RF)	AIP vs. PC	cross-validation	1b	0
Frøkjær2020	MRI (DWI)	1 reader; not	3D Slicer	tenfold cross-validation	Bayes	CP vs.	cross-	1b	0

		mentioned	Radiomics	forward selection procedure	classifier	normal tissue	validation		
Hu2021	MRI (T2WI)	2 readers; not mentioned	IBEX on MATLAB	(1) ANOVA; (2) absolute shrinkage and selection operator (LASSO)	logistic regression (LR)	AP (recurrence)	randomly split-sample	2a	II
Iranmahboob2017	MRI (ADC)	2 readers; blinded	FireVoxel	None	logistic regression (LR)	AP (progression)	the same data	1a	0
Li2021	CT (arterial)	1 reader; not mentioned	MaZda	samples t test or the Mann-Whitney U test	logistic regression (LR)	AIP vs. PC	the same data	1a	0
Li2022	CT (portal)	2 readers; blinded	Pyradiomics	(1) variance analysis; (2) Spearman's correlation analysis; (3) least absolute shrink- age and selection operator method (LASSO) logistic regression algorithm	logistic regression (LR)	AIP vs. PC	cross-validation	1b	0
Lin2019	CT (arterial, portal, and delay)	2 readers; blinded	Artificial Intelligent Kit	samples t test or the Mann-Whitney U test	logistic regression (LR)	AIP vs. PC	the same data	1a	0
Lin2020	MRI (portal)	2 readers; blinded	IBEX on MATLAB	(1) samples t test or the Mann-Whitney U test; (2) Boruta algorithm	support vector machine (SVM)	AP (severity)	randomly split-sample	2a	II
Liu2021	PET/CT (FDG)	2 readers; blinded	3D Slicer Radiomics	support vector machine recursive feature elimination (SVM-RFE)	support vector machine (SVM)	AIP vs. PC	cross-validation	1b	0
Liu2022	MRI (T1WI, T2WI, DWI, and ADC)	2 readers; blinded	Pyradiomics	(1) minimum-redundancy maximum-relevance (mRMR) algorithm; (2) least absolute shrinkage and selection operator (LASSO)	logistic regression (LR)	MFP vs. PC	randomly split-sample	2a	II
Ma2022	CT (arterial,	2 readers;	Pyradiomics	(1) preserve features	logistic	CP vs. PC	cross-	1b	0

	and portal)	blinded		with good consistence; (2) univariate Wilcoxon rank–sum test to retain P < 0.00005 (adjusted P value by Bonferroni method) features; (3) correlation analysis to remove the features with correlation >0.9; (4) the least absolute shrinkage and selection operator algorithm (LASSO)	regression (LR)		validation		
Mashayekhi2020	CT (portal)	2 readers; not mentioned	in-house software on MATLAB	Wilcoxon rank sum test	one-vs-one (OVO) IsoSVM classifier	CP vs. recurrent AP vs. functional abdominal pain	cross-validation	1b	0
Park2020	CT (arterial, and portal)	4 readers; confirmed by 4 readers; not mentioned	NR	minimum-redundancy maximum-relevancy feature selection	random forest (RF)	AIP vs. PC	randomly split-sample	2a	II
Peng2020	MRI (T2WI)	1 reader; not mentioned	Omni-Kinetics	ANOVA, LSD-*t* test or Kruskal-Walls test	logistic regression (LR)	MFP vs. PC; MFP vs. SPT + pNET	the same data	1a	0
Ren2019	CT (arterial, and portal)	2 readers; not mentioned	AnalysisKit	(1) minimum redundancy maximum relevance (MRMR); (2) stepwise-backward multivariate logistic regression	logistic regression (LR)	MFP vs. PC	randomly split-sample	2a	II
Ren2020	CT (unenhanced)	NR	NR	(1) synthetic minority oversampling technique (SMOTE); (2) Mann–Whitney U test; (3) minimum redundancy maximum relevance (MRMR)	random forest (RF)	MFP vs. PC	randomly split-sample	2a	II
Ren2021	MRI (ADC)	2 readers; not	ImageJ	Mann–Whitney *U* test	logistic	MFP vs. PC	the same	1a	0

		mentioned			regression (LR)		data		
Zhang2010	EUS	NR	in-house software on MATLAB	sequential forward selection	support vector machine (SVM)	PC vs. normal tissue or CP	randomly split-sample	2a	II
Zhang2019A	PET/CT (FDG)	1 reader; blinded	in-house software on MATLAB	(1) Spearman's rank correlation coefficient (Spearman); (2) minimum redundancy maximum relevance (MRMR); (3) and support vector machine recursive feature elimination (SVM-RFE)	(1) random forest (RF); (2) adaptive boosting (Adaboost); (3) support vector machine with the Gaussian radial basis function kernel function (RBF SVM); (4) SVM with the linear kernel function (Linear SVM)	AIP vs. PC	cross-validation	1b	0
Zhang2019B	PET/CT (FDG)	NR	in-house software on MATLAB	(1) Fisher method; (2) sequence forward floating selection algorithm support vector machine (SFSS-SVM)	support vector machine (SVM)	AIP vs. PC	cross-validation	1b	0
Zhang2019C	CT (arterial)	NR	AnalysisKit	least absolute shrink-age and selection operator method (LASSO)	logistic regression (LR)	MFP vs. PC	cross-validation	1b	I
Zhou2021	MRI (T2WI)	2 readers;	IBEX on	(1) samples t test or the	support	AP (extra-	randomly	2a	II

		blinded	MATLAB	Mann-Whitney U test; (2) least absolute shrinkage and selection operator (LASSO).	vector machine (SVM)	pancreatic necrosis)	split-sample		
Zhu2013	EUS	NR	in-house software on MATLAB	sequential forward selection (SFS) algorithm	support vector machine (SVM)	CP vs. PC	randomly split-sample	2a	II
Zhu2015	EUS	NR; blinded	in-house software on MATLAB	(1) distance between class methods; (2) sequential forward selection algorithm.	support vector machine (SVM)	AIP vs. CP	randomly split-sample	2a	II
Ziegelmayer2020	CT	2 readers + automatic method; not blinded	PyRadiomics	(1) Low variance features (below 0.1); (2) Spearman's correlation coefficient (above or below ± 0.9)	extremely randomized trees classifier	AIP vs. PC	Two-fold shuffle-split cross-validation	1b	I

Note: PC = pancreatic cancer, CP = chronic pancreatitis, AIP = autoimmune pancreatitis, MFP = mass-forming pancreatitis, AP = acute pancreatitis, SPT = solid-pseudopapillary tumor of pancreas, pNET = pancreatic neuroendocrine tumor.

Supplementary Table S12 RQS Rating per Study

Study	Chen2019	Cheng2018	Cui2021	Das2008	Deng2021	E2020	Frøkjær2020	Hu2021	Iranmahboob[illegible]	Li2021	Li2022	Lin2019	Lin2020	Liu2021	Liu2022	Ma2022	Mashayekhi2[illegible]	Park2020	Peng2020	Ren2019	Ren2020	Ren2021	Zhang2010	Zhang2019A	Zhang2019B	Zhang2019C	Zhou2021	Zhu2013	Zhu2015	Ziegelmayer2[illegible]
**Total 16 items (ideal score 36)**	16	4	4	9	15	5	9	11	-3	4	5	2	14	6	18	11	3	9	2	12	9	4	8	5	0	4	14	8	8	2
**Domain 1: protocol quality and stability in image and segmentation (0 to 5 points)**	2	2	2	1	2	2	1	2	2	1	2	2	2	2	2	2	2	2	1	2	1	2	1	1	0	1	2	1	1	1
**Protocol quality (2 points)**	1	1	1	1	1	1	1	1	1	1	1	1	1	1	1	1	1	1	1	1	1	1	1	1	0	1	1	1	1	0
**Multiple segmentations (1 point)**	1	1	1	0	1	1	0	1	1	0	1	1	1	1	1	1	1	1	0	1	0	1	0	0	0	0	1	0	0	1
**Test-retest (1 point)**	0	0	0	0	0	0	0	0	0	0	0	0	0	0	0	0	0	0	0	0	0	0	0	0	0	0	0	0	0	0
**Phantom study (1 point)**	0	0	0	0	0	0	0	0	0	0	0	0	0	0	0	0	0	0	0	0	0	0	0	0	0	0	0	0	0	0
**Domain 2: feature selection and validation (-8 to 8 points)**	5	-2	-2	5	6	-2	-2	5	-8	-2	-2	-2	5	-2	5	-2	-2	5	-2	5	5	-2	5	-2	-2	-2	5	5	5	-2
**Feature reduction or adjustment of multiple**	3	3	3	3	3	3	3	3	-3	3	3	3	3	3	3	3	3	3	3	3	3	3	3	3	3	3	3	3	3	3

**testing (-3 or 3 points)**																														
**Validation (-5, 2, 3, 4, or 5 points)**	2	-5	-5	2	3	-5	-5	2	-5	-5	-5	-5	2	-5	2	-5	-5	2	-5	2	2	-5	2	-5	-5	-5	2	2	2	-5
**Domain 3: biologic/clinical validation and utility (0 to 6 points)**	4	2	2	0	3	3	1	1	1	3	2	1	4	4	6	6	1	0	1	3	1	1	0	4	0	3	4	0	0	0
**Non-radiomics features (1 point)**	1	1	1	0	1	0	0	1	0	1	1	0	1	1	1	1	0	0	0	1	0	0	0	1	0	1	1	0	0	0
**Biologic correlations (1 point)**	1	1	1	0	0	1	1	0	1	0	1	1	1	1	1	1	1	0	1	0	1	1	0	1	0	0	1	0	0	0
**Comparison to “gold standard” (2 points)**	2	0	0	0	2	2	0	0	0	2	0	0	2	2	2	2	0	0	0	2	0	0	0	2	0	2	2	0	0	0
**Potential clinical utility (2 points)**	0	0	0	0	0	0	0	0	0	0	0	0	0	0	2	2	0	0	0	0	0	0	0	0	0	0	0	0	0	0
**Domain 4: model performance index (0 to 5 points)**	4	2	2	2	3	2	1	2	1	2	2	1	2	2	4	4	2	2	2	2	2	2	2	2	2	2	2	2	2	2
**Cut-off analysis (1 point)**	0	0	0	0	0	0	0	0	0	0	0	0	0	0	0	0	0	0	0	0	0	0	0	0	0	0	0	0	0	0
**Discrimination statistics (2 points)**	2	2	2	2	2	2	1	2	1	2	2	1	2	2	2	2	2	2	2	2	2	2	2	2	2	2	2	2	2	2
**Calibration statistics (2**	2	0	0	0	1	0	0	0	0	0	0	0	0	0	2	2	0	0	0	0	0	0	0	0	0	0	0	0	0	0

**points)**																														
**Domain 5: high level of evidence (0 to 8 points)**	0	0	0	0	0	0	7	0	0	0	0	0	0	0	0	0	0	0	0	0	0	0	0	0	0	0	0	0	0	0
**Prospective study (7 points)**	0	0	0	0	0	0	7	0	0	0	0	0	0	0	0	0	0	0	0	0	0	0	0	0	0	0	0	0	0	0
**Cost-effectiveness analysis (1 point)**	0	0	0	0	0	0	0	0	0	0	0	0	0	0	0	0	0	0	0	0	0	0	0	0	0	0	0	0	0	0
**Domain 6: Open science and data (0 to 4 points)**	1	0	0	1	1	0	1	1	1	0	1	0	1	0	1	1	0	0	0	0	0	1	0	0	0	0	1	0	0	1

Note: In case a score of at least one point for each item of RQS was obtained without minus points, it was considered to have basic adherence.

Supplementary Table S13 TRIPOD Adherence per Study

Study	Chen2019	Cheng2018	Cui2021	Das2008	Deng2021	E2020	Frøkjær2020	Hu2021	Iranmahboob201	Li2021	Li2022	Lin2019	Lin2020	Liu2021	Liu2022	Ma2022	Mashayekhi2020	Park2020	Peng2020	Ren2019	Ren2020	Ren2021	Zhang2010	Zhang2019A	Zhang2019B	Zhang2019C	Zhou2021	Zhu2013	Zhu2015	Ziegelmayer2020
**Overall**	20	17	16	11	19	19	16	17	16	15	19	15	20	18	23	19	16	16	13	17	16	14	13	17	12	12	18	10	12	12
**Title and Abstract**	1	0	0	1	1	1	2	1	0	0	0	0	1	1	2	0	0	0	0	0	1	0	1	1	0	0	0	0	0	0
**1**	0	0	0	0	0	0	1	0	0	0	0	0	0	0	1	0	0	0	0	0	0	0	0	0	0	0	0	0	0	0
**2**	1	0	0	1	1	1	1	1	0	0	0	0	1	1	1	0	0	0	0	0	1	0	1	1	0	0	0	0	0	0
**Introduction**	2	1	1	2	2	1	1	1	1	1	1	1	2	2	2	1	1	1	1	1	1	1	1	1	1	1	1	1	2	1
**3a**	1	1	1	1	1	1	1	1	1	1	1	1	1	1	1	1	1	1	1	1	1	1	1	1	1	1	1	1	1	1
**3b**	1	0	0	1	1	0	0	0	0	0	0	0	1	1	1	0	0	0	0	0	0	0	0	0	0	0	0	0	1	0
**Methods**	10	9	9	6	10	10	8	9	9	8	10	8	10	9	10	10	9	8	8	9	7	8	7	9	6	7	10	6	7	6
**4a**	1	1	1	1	1	1	1	1	1	1	1	1	1	1	1	1	1	1	1	1	1	1	1	1	1	1	1	1	1	1
**4b**	1	1	1	0	1	1	0	1	1	1	1	1	1	1	1	1	1	1	1	1	1	1	1	1	0	1	1	1	1	0
**5a**	1	1	1	1	1	1	1	1	1	1	1	1	1	1	1	1	1	1	1	1	1	1	1	1	1	1	1	1	1	1
**5b**	1	1	1	0	1	1	1	1	1	1	1	1	1	0	1	1	1	0	1	1	1	1	0	0	1	0	1	0	0	0
**5c, if relevant**	0	n/a	0	n/a	n/a	n/a	n/a	0	0	n/a	n/a	n/a	n/a	n/a	0	n/a	n/a	n/a	n/a	n/a	n/a	n/a	n/a	n/a	n/a	n/a	0	n/a	n/a	n/a
**6a**	1	1	1	1	1	1	1	1	1	1	1	1	1	1	1	1	1	1	1	1	1	1	1	1	1	1	1	1	1	1
**6b**	0	0	0	0	0	0	0	0	0	0	0	0	0	0	0	0	0	0	0	0	0	0	0	0	0	0	0	0	0	0
**7a**	1	1	1	0	1	1	1	1	1	1	1	0	1	1	1	1	1	1	1	1	0	1	1	1	0	0	1	0	0	1
**7b**	1	1	1	0	1	1	0	0	1	0	1	1	1	1	1	1	0	0	0	0	0	0	0	1	0	0	1	0	1	0
**8**	0	0	0	0	0	0	0	0	0	0	0	0	0	0	0	0	0	0	0	0	0	0	0	0	0	0	0	0	0	0
**9**	0	0	0	0	0	0	0	0	0	0	0	0	0	0	0	0	0	0	0	0	0	0	0	0	0	0	0	0	0	0
**10a**	1	1	1	1	1	1	1	1	1	1	1	1	1	1	1	1	1	1	1	1	0	1	0	1	0	1	1	0	0	0
**10b**	1	0	0	1	1	1	1	1	0	0	1	0	1	1	1	1	1	1	0	1	1	0	1	1	1	1	1	1	1	1
**10d**	1	1	1	1	1	1	1	1	1	1	1	1	1	1	1	1	1	1	1	1	1	1	1	1	1	1	1	1	1	1
**11, if done**	n/a	n/a	n/a	n/a	n/a	n/a	n/a	n/a	n/a	n/a	n/a	n/a	n/a	n/a	n/a	n/a	n/a	n/a	n/a	n/a	n/a	n/a	n/a	n/a	n/a	n/a	n/a	n/a	n/a	n/a

**Results**	4	4	3	0	4	4	2	3	3	3	5	3	4	3	6	5	3	4	2	4	5	3	1	3	2	2	4	1	1	3
**13a**	1	1	0	0	1	1	0	0	1	0	1	1	1	0	1	0	1	1	0	1	1	1	0	0	0	0	1	0	0	1
**13b**	1	1	1	0	1	1	1	1	1	0	1	1	1	1	1	1	1	1	1	1	1	1	0	1	0	1	1	0	0	1
**14a**	1	1	1	0	1	1	1	1	1	1	1	1	1	1	1	1	1	1	1	1	1	1	0	1	1	1	1	0	0	0
**14b, if done**	n/a	0	n/a	n/a	0	n/a	n/a	1	n/a	n/a	n/a	n/a	n/a	n/a	n/a	n/a	0	n/a	n/a	n/a	0	n/a	n/a	n/a	n/a	n/a	n/a	n/a	n/a	n/a
**15a**	0	0	0	0	0	0	0	0	0	1	1	0	0	0	1	1	0	0	0	0	1	0	0	0	0	0	0	0	0	0
**15b**	0	0	0	0	0	0	0	0	0	0	0	0	0	0	1	1	0	0	0	0	0	0	0	0	0	0	0	0	0	0
**16**	1	1	1	0	1	1	0	1	0	1	1	0	1	1	1	1	0	1	0	1	1	0	1	1	1	0	1	1	1	1
**Discussion**	3	3	3	2	2	3	3	3	3	3	3	3	3	3	3	3	3	3	2	3	2	2	3	3	3	2	3	2	2	2
**18**	1	1	1	1	1	1	1	1	1	1	1	1	1	1	1	1	1	1	1	1	1	1	1	1	1	1	1	1	1	1
**19b**	1	1	1	1	1	1	1	1	1	1	1	1	1	1	1	1	1	1	1	1	1	1	1	1	1	1	1	1	1	1
**20**	1	1	1	0	0	1	1	1	1	1	1	1	1	1	1	1	1	1	0	1	0	0	1	1	1	0	1	0	0	0
**Validation**	2	n/a	n/a	0	2	n/a	n/a	0	n/a	n/a	n/a	n/a	2	n/a	1	n/a	n/a	0	n/a	0	0	n/a	0	n/a	n/a	n/a	2	0	0	n/a
**Model type**	2a	1a	1a	2a	2b	1b	1b	2a	1a	1a	1b	1a	2a	1b	2a	1b	1b	2a	1a	2a	2a	1a	2a	1b	1b	1b	2a	2a	2a	1b
**10c**	0	n/a	n/a	0	0	n/a	n/a	0	n/a	n/a	n/a	n/a	0	n/a	0	n/a	n/a	0	n/a	0	0	n/a	0	n/a	n/a	n/a	0	0	0	n/a
**10e, if done**	n/a	n/a	n/a	n/a	n/a	n/a	n/a	n/a	n/a	n/a	n/a	n/a	n/a	n/a	n/a	n/a	n/a	n/a	n/a	n/a	n/a	n/a	n/a	n/a	n/a	n/a	n/a	n/a	n/a	n/a
**12**	1	n/a	n/a	0	1	n/a	n/a	0	n/a	n/a	n/a	n/a	1	n/a	1	n/a	n/a	0	n/a	0	0	n/a	0	n/a	n/a	n/a	1	0	0	n/a
**13c**	1	n/a	n/a	0	1	n/a	n/a	0	n/a	n/a	n/a	n/a	1	n/a	0	n/a	n/a	0	n/a	0	0	n/a	0	n/a	n/a	n/a	1	0	0	n/a
**17, if done**	n/a	n/a	n/a	n/a	n/a	n/a	n/a	n/a	n/a	n/a	n/a	n/a	n/a	n/a	n/a	n/a	n/a	n/a	n/a	n/a	n/a	n/a	n/a	n/a	n/a	n/a	n/a	n/a	n/a	n/a
**19a**	0	n/a	n/a	0	0	n/a	n/a	0	n/a	n/a	n/a	n/a	0	n/a	0	n/a	n/a	0	n/a	0	0	n/a	0	n/a	n/a	n/a	0	0	0	n/a

Note: 1 = reported, 0 = not reported. The basic adherence of items in TRIPOD checklist and IBSI guideline was defined as those have been reported.

Supplementary Table S14 Pre-processing Steps Performed in Each Study

Study	Chen2019	Cheng2018	Cui2021	Das2008	Deng2021	E2020	Frøkjær2020	Hu2021	Iranmahboob201[illegible]	Li2021	Li2022	Lin2019	Lin2020	Liu2021	Liu2022	Ma2022	Mashayekhi2020	Park2020	Peng2020	Ren2019	Ren2020	Ren2021	Zhang2010	Zhang2019A	Zhang2019B	Zhang2019C	Zhou2021	Zhu2013	Zhu2015	Ziegelmayer2020
Intensity normalization	Y	N	N	N	Y	N	N	N	N	N	N	N	Y	Y	Y	Y	N	N	N	N	Y	N	N	Y	Y	N	Y	N	N	Y
Segmentation method	Y	Y	Y	N	Y	Y	Y	Y	Y	Y	Y	Y	Y	Y	Y	Y	Y	Y	Y	Y	N	Y	N	Y	N	N	Y	N	N	Y
Image interpolation/iso-voxel resampling	Y	N	N	N	N	N	N	N	N	N	N	N	N	Y	N	Y	N	N	N	N	Y	N	N	Y	Y	N	N	N	N	Y
Grey-level discretization	Y	N	N	N	N	N	N	N	N	N	N	N	N	Y	N	N	N	N	N	N	Y	N	N	Y	Y	N	N	N	N	Y
Image filtering	N	N	N	Y	Y	N	Y	N	N	N	Y	N	N	N	N	Y	N	N	N	N	N	N	N	N	Y	N	N	N	N	Y
IBSI compliance of radiomics extraction software	Y	N	N	N	Y	N	Y	Y	N	Y	Y	N	Y	Y	Y	Y	N	N	N	N	N	N	N	N	N	N	Y	N	N	Y
Robustness of imaging biomarkers assessment	Y	N	N	N	Y	Y	N	Y	Y	N	Y	N	Y	N	Y	Y	Y	N	N	N	N	Y	N	N	N	N	Y	N	N	N

Note: Y = Yes, reported; N = No, not reported. The basic adherence of items in TRIPOD checklist and IBSI guideline was defined as those have been reported.

Supplementary Table S15 QUADAS-2 Assessment per Study

Study	Chen2019	Cheng2018	Cui2021	Das2008	Deng2021	E2020	Frøkjær2020	Hu2021	Iranmahboob2017	Li2021	Li2022	Lin2019	Lin2020	Liu2021	Liu2022	Ma2022	Mashayekhi2020	Park2020	Peng2020	Ren2019	Ren2020	Ren2021	Zhang2010	Zhang2019A	Zhang2019B	Zhang2019C	Zhou2021	Zhu2013	Zhu2015	Ziegelmayer2020
**Risk of bias**																														
**Patient Selection**	L	H	L	H	L	L	H	L	L	L	L	L	L	H	L	L	L	H	L	L	L	L	H	H	L	H	L	H	H	H
**Index Test**	L	H	H	H	L	H	H	L	H	H	H	H	L	H	L	H	H	H	H	L	H	H	H	H	H	H	L	H	H	H
**Reference Standard**	L	L	L	L	L	L	L	L	L	L	L	L	L	L	L	H	L	L	L	L	L	L	L	L	L	L	L	L	L	L
**Flow and Timing**	L	L	L	U	U	U	U	U	U	L	U	U	L	U	U	U	U	U	U	L	U	L	U	U	U	U	L	U	U	U
**Application concern**																														
**Patient Selection**	L	H	L	U	L	L	U	L	L	L	L	L	L	U	L	L	L	H	U	L	L	L	U	U	L	U	L	U	U	U
**Index Test**	L	H	L	H	L	H	H	L	H	H	H	H	L	H	L	H	H	H	H	L	H	H	H	H	H	H	L	H	H	H
**Reference Standard**	L	L	L	L	L	L	L	L	L	L	L	L	L	L	L	H	L	L	L	L	L	L	L	L	L	L	L	L	L	L

Note:

Supplementary Table S16 Model Metrics of Studies Included in Meta-analysis

Study	Imaging modality	Predictor	Reference standard	Dataset	TP	FP	FN	TN	AUC (95%CI)	Inclusion with comment
**Autoimmune pancreatitis vs. Pancreatic cancer by CT**<br>P for autoimmune pancreatitis, N for pancreatic cancer; 6 studies, 5/8 available datasets, 191/421 events/sample size										
E2020	CT (non-enhanced, arterial, portal)	Radiomics	Clinical criteria or histology; Histology	Training	42	2	3	49	0.977 (0.924-0.997)	Yes.
Li2021	CT (arterial)	Radiomics + objective assessment	Clinical criteria or histology; Histology	Training	41	4	9	56	0.937 (0.894-0.979)	Yes.
Li2022	CT (portal)	Radiomics	Clinical criteria; Histology	Training	40	4	2	51	0.97 (0.92-0.99)	Yes.
Lin2019	CT (arterial, portal, delay)	Radiomics	Clinical criteria; Histology	Training	23	1	2	30	0.973	Yes.
Park2020	CT (arterial, portal)	Radiomics	Clinical criteria or histology; Histology	Training	NR	NR	NR	NR	NR	No, unavailable data.
Park2020	CT (arterial, portal)	Radiomics	Clinical criteria or histology; Histology	Validation	26	0	3	33	0.98 (0.94-1.00)	Yes.
Ziegelmayer2020	CT (portal)	Radiomics	Clinical criteria or histology; Histology	Training	NR	NR	NR	NR	NR	No, unavailable data.
Ziegelmayer2020	CT (portal)	Radiomics	Clinical criteria or histology; Histology	Validation	NR	NR	NR	NR	0.80 (0.79-0.81)	No, unavailable data.
**Autoimmune pancreatitis vs. Pancreatic cancer by PET**<br>P for autoimmune pancreatitis, N for pancreatic cancer; 4 studies, 3/4 available datasets, 143/337 events/sample size										
Cheng2018	PET/CT (FDG)	Radiomics + objective assessment	Histology; Histology	Training	42	6	8	58	0.95 (0.88–0.98)	Yes.
Liu2021	PET/CT (FDG)	Radiomics + clinical	Clinical criteria or histology; Histology	Training	41	2	7	62	0.9657 (0.9581-0.9733)	Yes.
Zhang2019A	PET/CT (FDG)	Radiomics	Histology; Histology	Training	38	9	7	57	0.93 (0.92-0.94)	No, overlapping data.
Zhang2019B	PET/CT (FDG)	Radiomics	Histology; Histology	Training	40	7	5	59	0.9218	Yes, better performance.
**Mass-forming pancreatitis vs. Pancreatic cancer by CT**<br>P for mass-forming pancreatitis, N for pancreatic cancer; 3 studies, 1/4 available datasets, 30/119 events/sample size										
Ren2019	CT (arterial, portal)	Radiomics	Histology; Histology	Training	19	4	1	45	0.92	No, overlapping data.

		+ objective assessment								
Ren2019	CT (arterial, portal)	Radiomics + objective assessment	Histology; Histology	Validation	9	6	12	4	0.90	No, overlapping data.
Ren2020	CT (non-enhanced)	Radiomics	Histology; Histology	Training	28	6	2	73	0.98 (0.97-1.00)	Yes, better performance.
Zhang2019C	CT (arterial)	Radiomics + objective assessment	Histology; Histology	Training	20	8	1	39	0.944 (0.860-0.985)	No, overlapping data.
**Mass-forming pancreatitis vs. Pancreatic cancer by MRI** P for mass-forming pancreatitis, N for pancreatic cancer; 4 studies, 5/6 available datasets, 101/320 events/sample size										
Deng2021	MRI (T1WI, T2WI, DCE)	Radiomics	Histology; Histology	Training	13	1	0	50	0.997 (0.990-1.000)	Yes.
Deng2021	MRI (T1WI, T2WI, DCE)	Radiomics	Histology; Histology	Validation	17	1	2	44	0.962 (0.907-1.000)	Yes.
Liu2022	MRI (T1WI, T2WI, DWI, ADC)	Radiomics + clinical	Histology; Histology	Training	30	3	4	35	0.973 (0.904–0.997)	Yes.
Liu2022	MRI (T1WI, T2WI, DWI, ADC)	Radiomics + clinical	Histology; Histology	Validation	12	1	2	15	0.960 (0.817–0.998)	Yes.
Peng2020	MRI (T2WI)	Radiomics	Histology; Histology	Training	18	12	3	57	0.899	Yes.
Ren2021	MRI (ADC)	Radiomics	Clinical criteria or histology; Histology	Training	NR	NR	NR	NR	0.92	No, unavailable data.

Note:

Supplementary Table S17 Diagnostic performance of meta-analyzed clinical questions regardless of imaging modality

**Clinical question**	**AIP vs. PC by CT and PET**	**AIP vs. PC by CT**	**MFP vs. PC by CT and MRI**	**MFP vs. PC by MRI**
Number of studies	10	6	7	4
Number of available datasets	8/12	5/8	6/10	5/6
Events/sample size	334/758	191/421	131/439	101/320
Pooled analysis				
DOR (95%CI)	118.13 (65.25-213.89)	189.63 (79.65-451.48)	133.37 (45.86-387-83)	135.70 (36.17-509.13)
P-value for DOR	< 0.001	< 0.001	< 0.001	< 0.001
Sensitivity (95% CI)	0.88 (0.84-0.92)	0.90 (0.84-0.94)	0.90 (0.83-0.95)	0.90 (0.81-0.95)
Specificity (95% CI)	0.94 (0.91-0.96)	0.95 (0.92-0.97)	0.93 (0.87-0.97)	0.94 (0.86-0.98)
PLR (95% CI)	14.60 (9.54-22.34)	19.01 (10.51-34.40)	13.66 (6.75-27.63)	15.00 (5.94-37.92)
NLR (95% CI)	0.12 (0.09-0.17)	0.10 (0.06-0.17)	0.10 (0.06-0.18)	0.11 (0.06-0.56)
AUC (95% CI)	0.96 (0.94-0.98)	0.97 (0.95-0.98)	0.95 (0.93-0.97)	0.95 (0.93-0.96)
Heterogeneity				
Higgins I^2 test (%)	71.82%	83.26%	96.33%	97.28%
Cochran's Q test (*P*-value)	< 0.01	<0.01	< 0.01	<0.01
Publication bias				
Egger's test (*P*-value)	0.002	0.060	0.060	0.050
Begg's test (*P*-value)	0.019	0.221	0.065	0.221
Deeks'test (*P*-value)	0.057	0.226	0.646	0.538
Trim and fill method				
Number of missing datasets	3	2	3	2
Adjusted DOR (95%CI)	85.57 (46.40-157.83)	135.11 (64.40-283.74)	52.93 (19.67-142.42)	53.89 (15.95-182.00)
Level of Evidence	Weak	Weak	Weak	Weak

Note:

Supplementary Table S18 Subgroup Analysis of Study Quality according to Study Characteristics

Subgroup	Studies, n	RQS rating, mean ± SD	P value	TRIPOD adherence rate, mean ± SD	P value	IBSI adherence rate, mean ± SD	P value
Journal type							
Imaging	16	18.63 ± 12.34%	0.424	65.13 ± 11.56%	0.056	36.69 ± 22.71%	0.928*
Non-imaging	14	22.64 ± 14.80%		56.86 ± 11.07%		37.64 ± 32.55%	
First authorship							
Radiologist	24	21.46 ± 14.34%	0.455	62.33 ± 11.77%	0.335	38.12 ± 26.60%	0.697
Non-radiologist	6	16.67 ± 9.07%		57.00 ± 12.55%		33.17 ± 32.05%	
Biomarker							
Diagnostic	24	18.79 ± 12.02%	0.168	60.54 ± 12.80%	0.514	34.50 ± 27.62%	0.298
Prognostic	6	27.33 ± 17.76%		64.17 ± 7.47%		47.67 ± 25.09%	
Imaging Modality							
CT	13	18.38 ± 12.02%	0.193	64.62 ± 11.31%	0.190	38.54 ± 30.67%	0.054**
EUS	4	22.75 ± 1.50%		49.75 ± 13.38%		3.5 ± 7.0%	
MRI	9	27.00 ± 17.64%		61.44 ± 11.06%		44.44 ± 17.98%	
PET	4	10.50 ± 7.42%		61.50 ± 11.33%		49.75 ± 24.73%	

Note: * Mann-Whitney's U-test. ** Kruskal-Wallis *H* test. The Bonferroni method was used for post-hoc correction ($p = 0.05/6 = 0.008$). Other comparisons were performed by student *t* test for intergroup differences, and one-way analysis of variance for multiple comparisons.

Supplementary Table S19 Correlation between Ideal Percentage of RQS, TRIPOD Adherence Rate, Sample Size and Impact Factor

**Correlation**	**Number of studies**	**Method**	**r**	**P value**
Ideal percentage of RQS X sample size	30	Spearman correlation test	0.456	**0.011**
Ideal percentage of RQS X impact factor	23	Spearman correlation test	0.329	0.125
TRIPOD adherence rate X sample size	30	Spearman correlation test	-0.179	0.345
TRIPOD adherence rate X impact factor	23	Spearman correlation test	-0.275	0.204
IBSI adherence rate X sample size	30	Spearman correlation test	0.055	> 0.999
IBSI adherence rate X impact factor	23	Spearman correlation test	-0.152	> 0.999

Note: Spearman correlation tests were used because of the non-normal distribution of impact factor and sample size.

Supplementary Figure S1 Forrest Plot of Diagnostic Odds Radio

(a) autoimmune pancreatitis vs. pancreatic cancer by CT and PET, (b) autoimmune pancreatitis vs. pancreatic cancer by CT, (c) mass-forming pancreatitis vs. pancreatic cancer by CT and MRI, (d) mass-forming pancreatitis vs. pancreatic cancer by MRI.

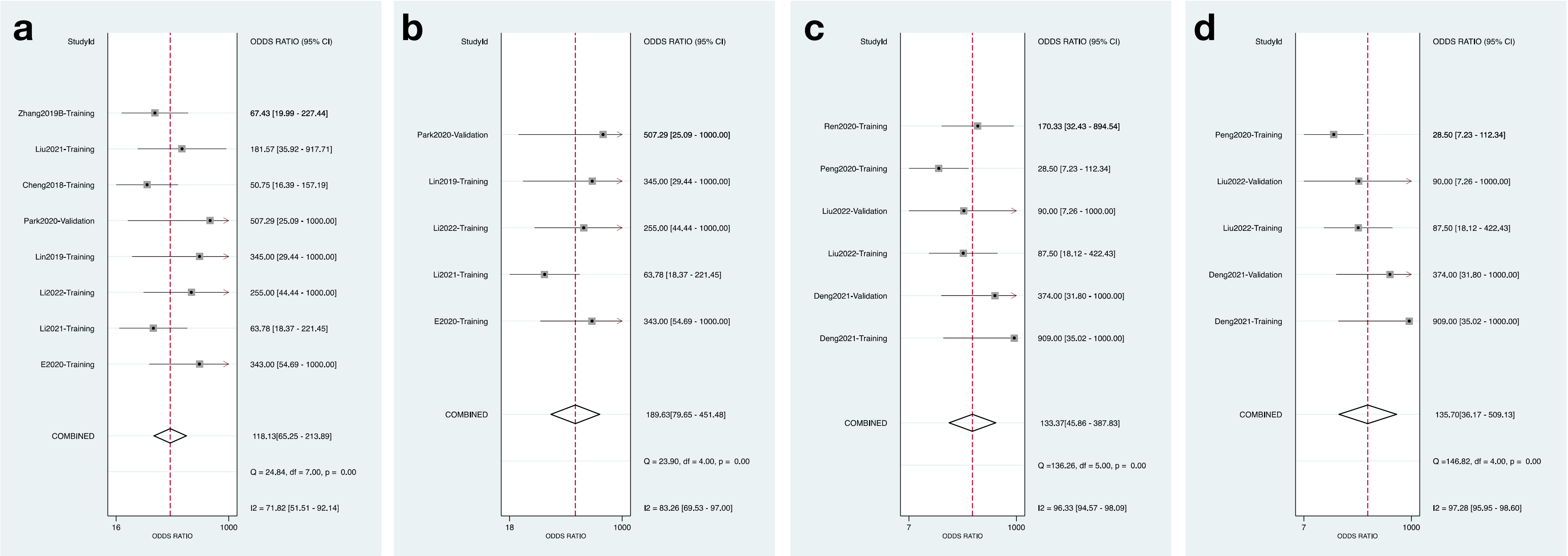

Supplementary Figure S2 Forrest Plot of Pooled Sensitivity
(a) autoimmune pancreatitis vs. pancreatic cancer by CT and PET, (b) autoimmune pancreatitis vs. pancreatic cancer by CT, (c) mass-forming pancreatitis vs. pancreatic cancer by CT and MRI, (d) mass-forming pancreatitis vs. pancreatic cancer by MRI.

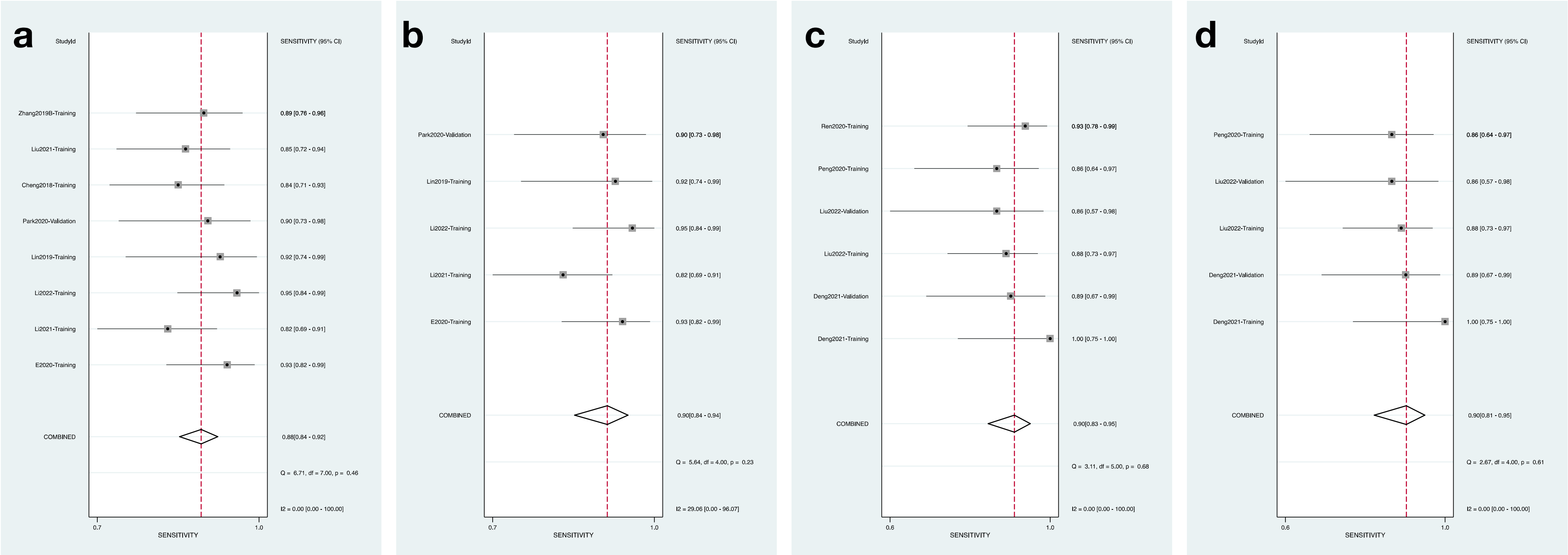

Supplementary Figure S3 Forrest Plot of Pooled Specificity
(a) autoimmune pancreatitis vs. pancreatic cancer by CT and PET, (b) autoimmune pancreatitis vs. pancreatic cancer by CT, (c) mass-forming pancreatitis vs. pancreatic cancer by CT and MRI, (d) mass-forming pancreatitis vs. pancreatic cancer by MRI.

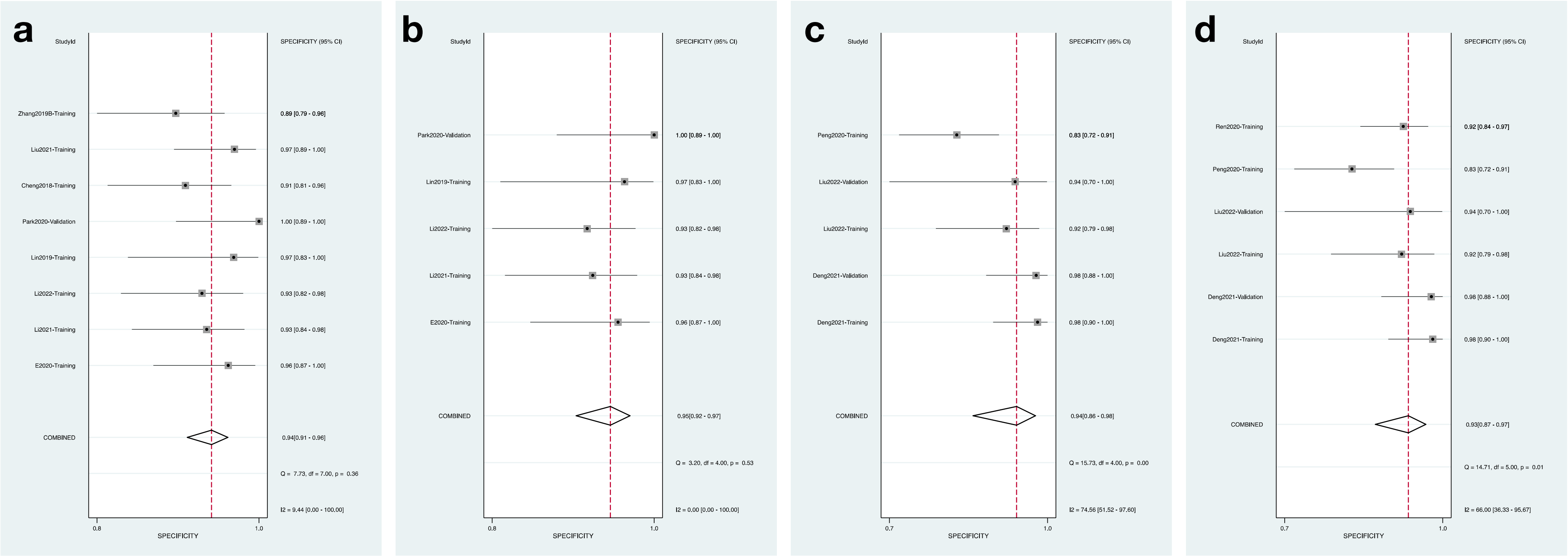

Supplementary Figure S4 Forrest Plot of Pooled Positive Likelihood Ratio
(a) autoimmune pancreatitis vs. pancreatic cancer by CT and PET, (b) autoimmune pancreatitis vs. pancreatic cancer by CT, (c) mass-forming pancreatitis vs. pancreatic cancer by CT and MRI, (d) mass-forming pancreatitis vs. pancreatic cancer by MRI.

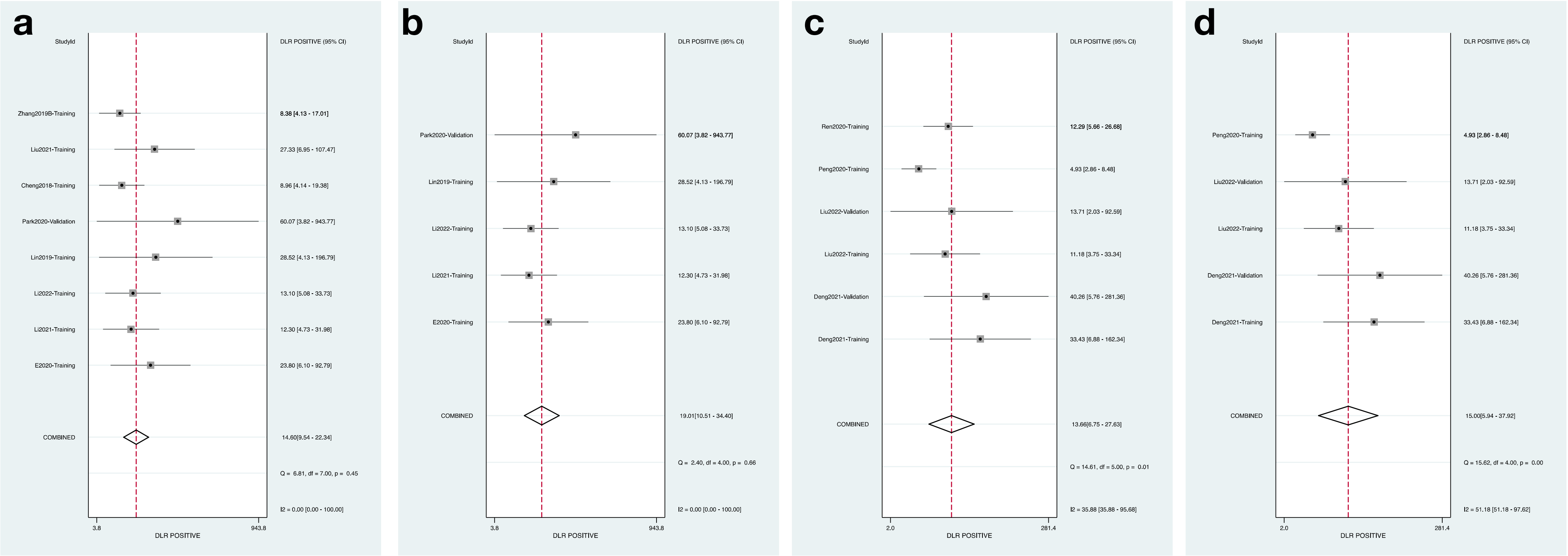

Supplementary Figure S5 Forrest Plot of Pooled Negative Likelihood Ratio

(a) autoimmune pancreatitis vs. pancreatic cancer by CT and PET, (b) autoimmune pancreatitis vs. pancreatic cancer by CT, (c) mass-forming pancreatitis vs. pancreatic cancer by CT and MRI, (d) mass-forming pancreatitis vs. pancreatic cancer by MRI.

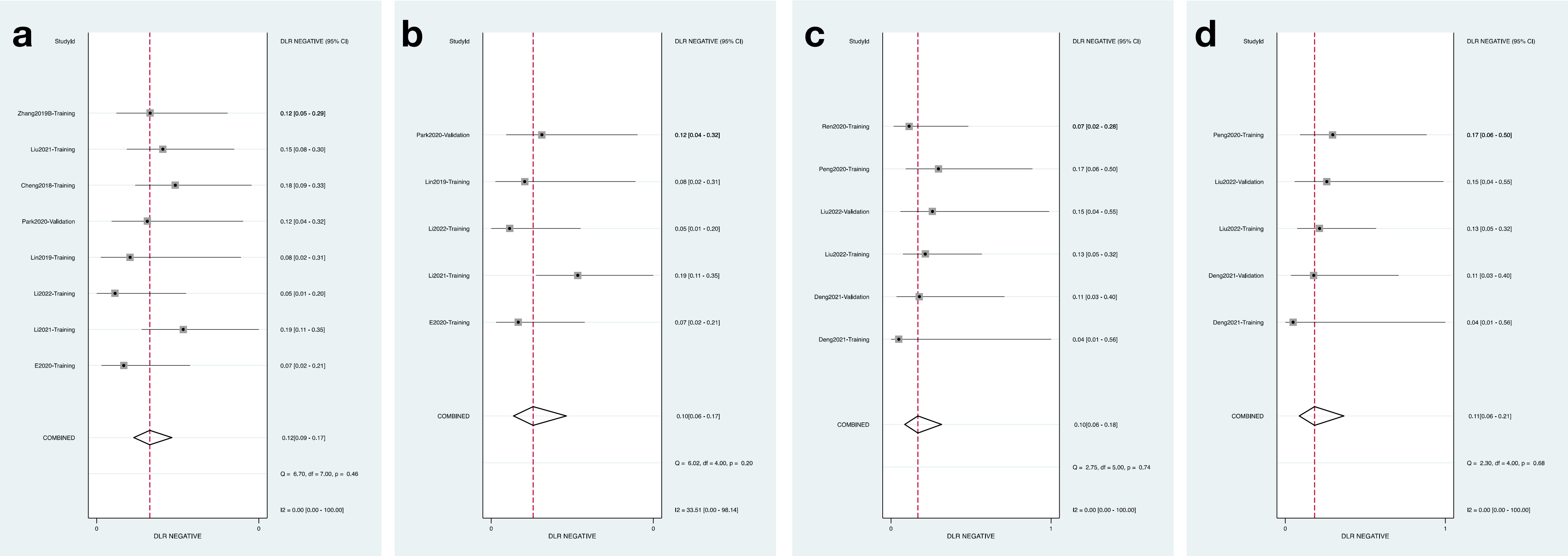

Supplementary Figure S6 HSROC Curve of the Model Performance

(a) autoimmune pancreatitis vs. pancreatic cancer by CT and PET, (b) autoimmune pancreatitis vs. pancreatic cancer by CT, (c) mass-forming pancreatitis vs. pancreatic cancer by CT and MRI, (d) mass-forming pancreatitis vs. pancreatic cancer by MRI.

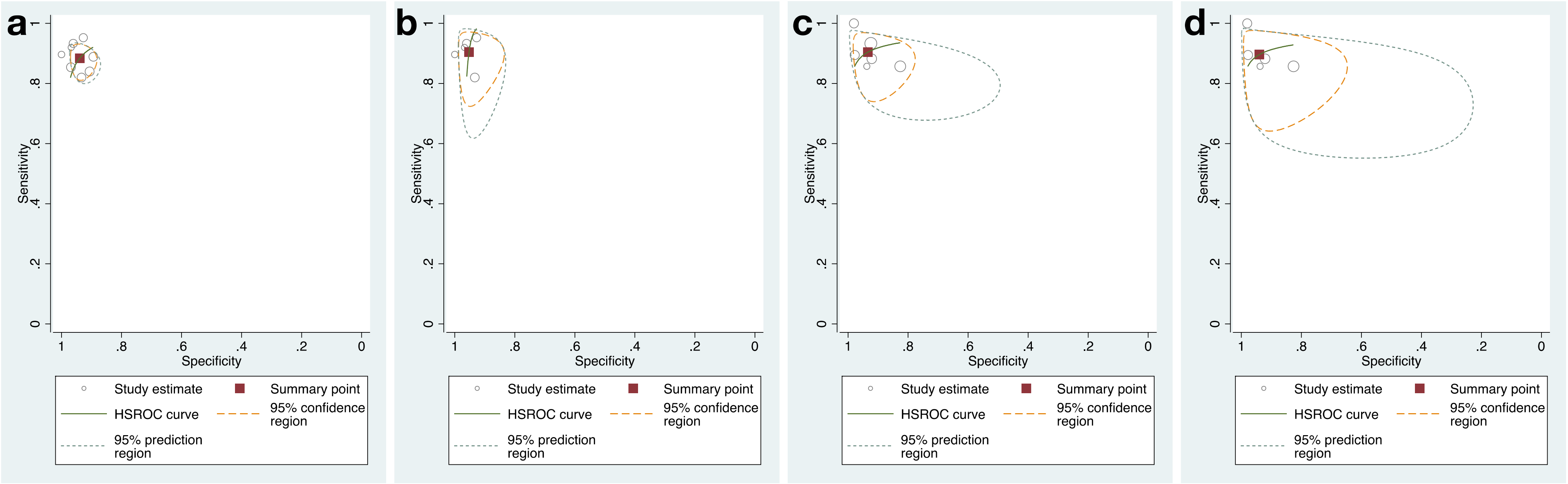

Supplementary Figure S7 Funnel plot of Studies Included in Meta-analysis
(a) autoimmune pancreatitis vs. pancreatic cancer by CT and PET, (b) autoimmune pancreatitis vs. pancreatic cancer by CT, (c) mass-forming pancreatitis vs. pancreatic cancer by CT and MRI, (d) mass-forming pancreatitis vs. pancreatic cancer by MRI.

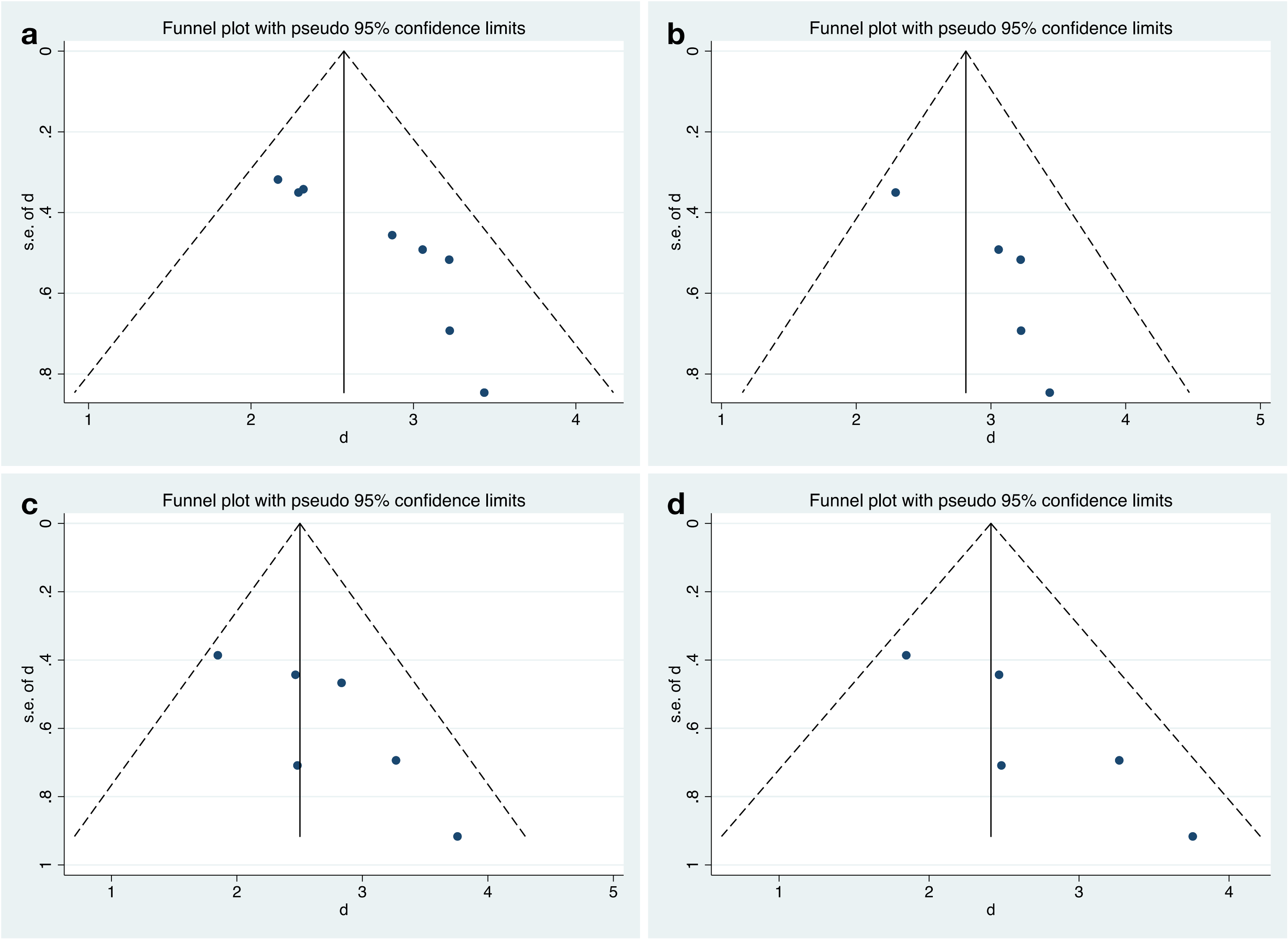

Supplementary Figure S8 Deeks Funnel Plot of Studies Included in Meta-analysis

(a) autoimmune pancreatitis vs. pancreatic cancer by CT and PET, (b) autoimmune pancreatitis vs. pancreatic cancer by CT, (c) mass-forming pancreatitis vs. pancreatic cancer by CT and MRI, (d) mass-forming pancreatitis vs. pancreatic cancer by MRI.

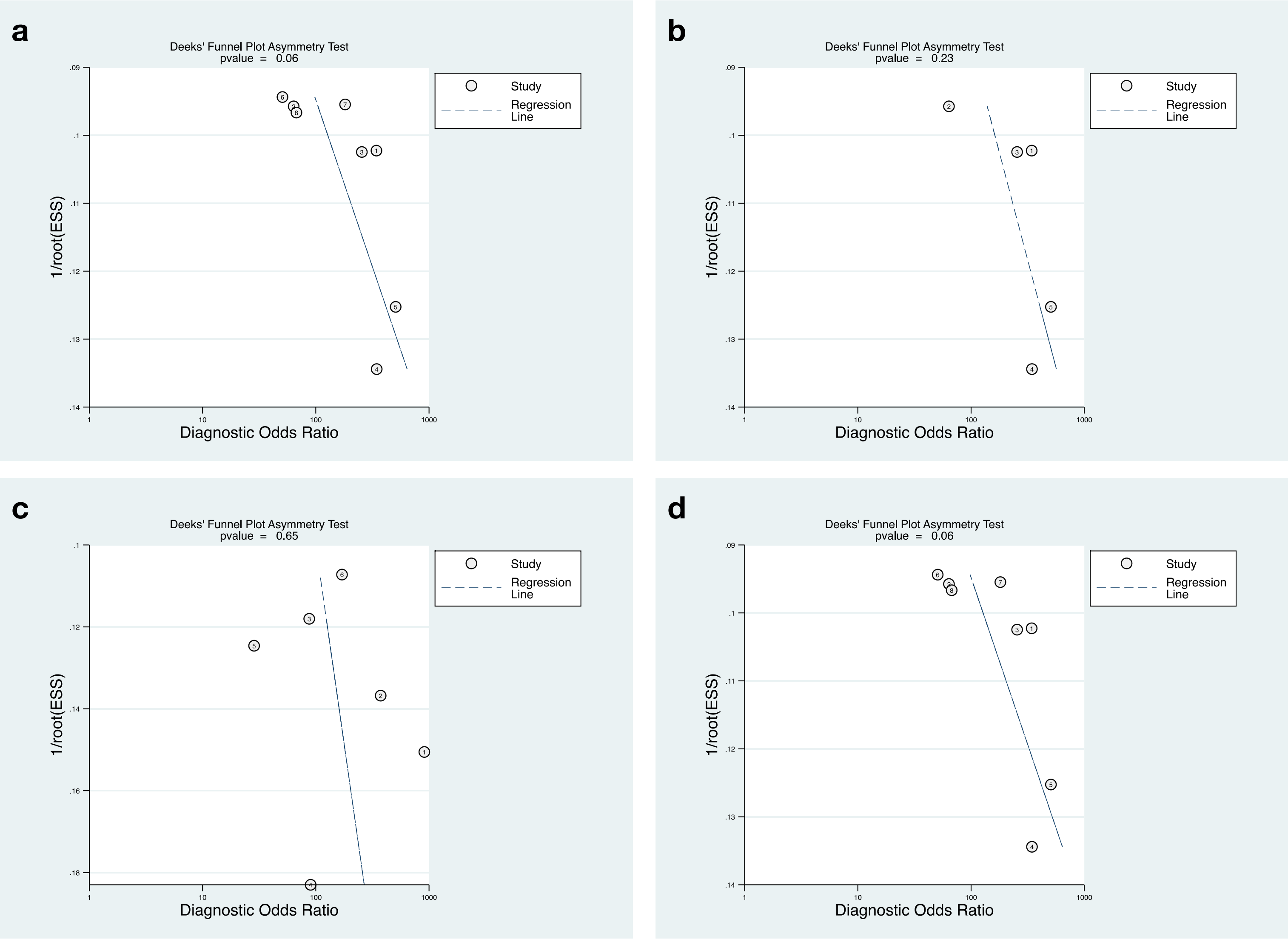

Insights Imaging (2022) Zhong J, Hu Y, Xing Y et al.

Supplementary Figure S9 Trim and Fill Analysis of Studies Included in Meta-analysis
(a) autoimmune pancreatitis vs. pancreatic cancer by CT and PET, (b) autoimmune pancreatitis vs. pancreatic cancer by CT, (c) mass-forming pancreatitis vs. pancreatic cancer by CT and MRI, (d) mass-forming pancreatitis vs. pancreatic cancer by MRI.

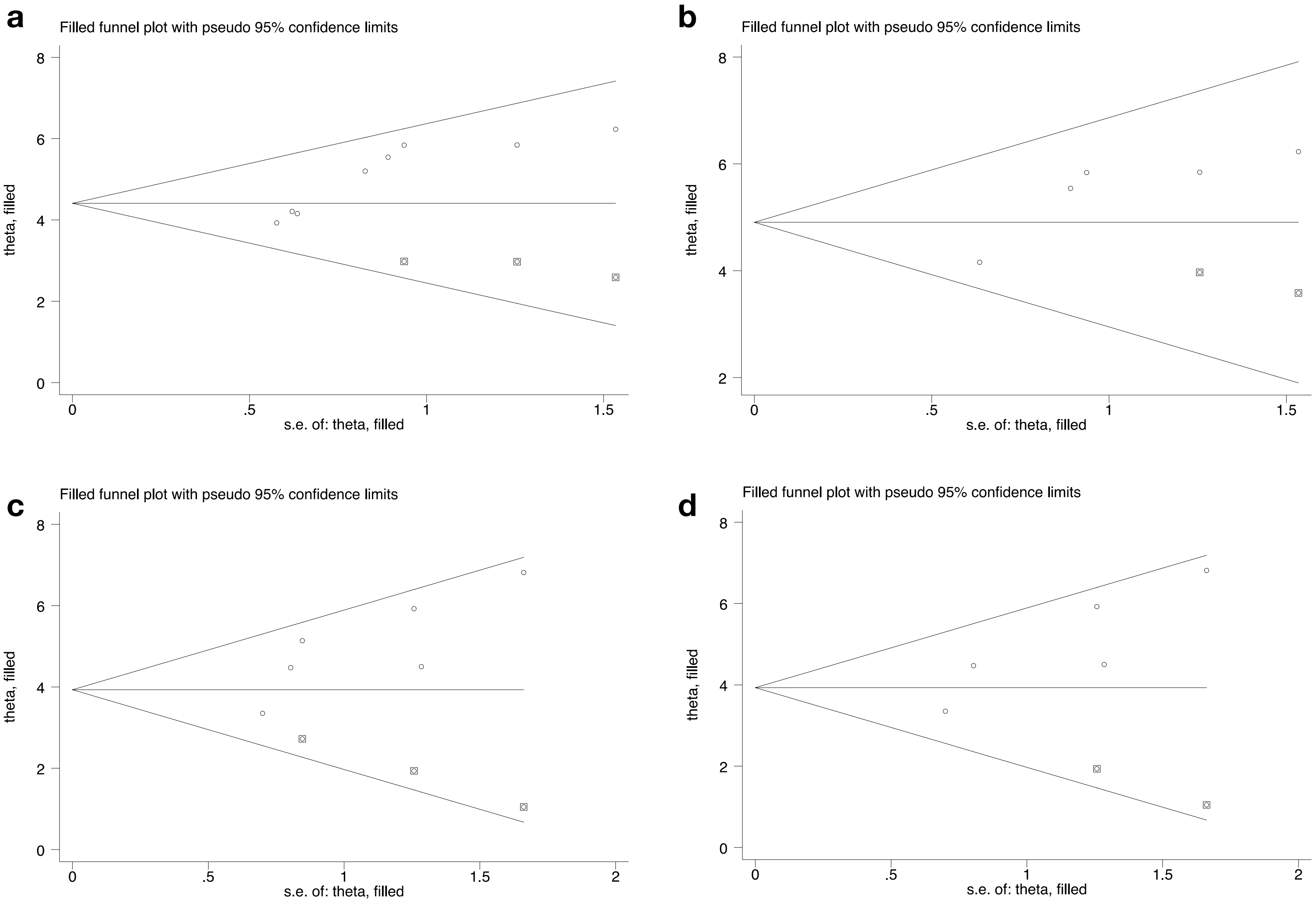

Supplement: Supplementary file 1 — Additional file 1: Supplementary Note S1. Review Protocol. Supplementary Note S2. Search Strategy and Study Selection. Supplementary Note S3. Consensus Reached during Data extraction and Quality Assessment. Supplementary Note S4. Data Synthesis and Analysis Methods. Supplementary Table S1. Data Extraction Sheet. Supplementary Table S2. Methodological Quality according to RQS Checklist. Supplementary Table S3. Reporting Completeness according to TRIPOD Statement. Supplementary Table S4. Pre-processing Steps according to IBSI Guideline. Supplementary Table S5. Risk of Bias and Concern on Application Assessment according to QUADAS-2 Tool. Supplementary Table S6. Types of Prediction Model Studies Covered by The TRIPOD Statement. Supplementary Table S7. Trials Classifications for Image Mining Tools Development Process. Supplementary Table S8. Category of Five Levels of Supporting Evidence of Meta-analyzes. Supplementary Table S9. Study Characteristics of Included Studies. Supplementary Table S10. PICOT of Included Studies. Supplementary Table S11. Radiomics Methodological Consideration of Included Studies. Supplementary Table S12. RQS Rating per Study. Supplementary Table S13. TRIPOD Adherence per Study. Supplementary Table S14. Pre-processing Steps Performed in Each Study. Supplementary Table S15. QUADAS-2 Assessment per Study. Supplementary Table S16. Model Metrics of Studies Included in Meta-analysis. Supplementary Table S17. Diagnostic performance of meta-analyzed clinical questions regardless of imaging modality. Supplementary Table S18. Subgroup Analysis of Study Quality according to Study Characteristics. Supplementary Table S19. Correlation between Ideal Percentage of RQS, TRIPOD Adherence Rate, Sample Size and Impact Factor. Supplementary Figure S1. Forrest Plot of Diagnostic Odds Radio. Supplementary Figure S2. Forrest Plot of Pooled Sensitivity. Supplementary Figure S3. Forrest Plot of Pooled Specificity. Supplementary Figure S4. Forrest Plot of Pooled Positiv [file 13244_2022_1279_MOESM1_ESM.pdf]
